# Increased thrombin generation in kidney transplant recipients with donor-specific antibodies directed against human leukocyte antigens

**DOI:** 10.3389/fimmu.2024.1407407

**Published:** 2024-10-25

**Authors:** Linda Lóczi, Réka P. Szabó, Rita Orbán-Kálmándi, Rebeka Hodossy-Takács, Anikó Szilvási, Zoltán Szalai, Gábor Nagy, Péter Antal-Szalmás, Balázs Nemes, Zsuzsa Bagoly

**Affiliations:** ^1^ Division of Clinical Laboratory Sciences, Department of Laboratory Medicine, Faculty of Medicine, Kálmán Laki Doctoral School, University of Debrecen, Debrecen, Hungary; ^2^ Hungarian Research Network-University of Debrecen (HUN-REN-DE) Cerebrovascular Research Group, Debrecen, Hungary; ^3^ Division of Nephrology, Department of Internal Medicine, University of Debrecen, Debrecen, Hungary; ^4^ Transplantation Immunogenetics Laboratory, Hungarian National Blood Transfusion Service, Budapest, Hungary; ^5^ Division of Organ Transplantation, Department of Surgery, Faculty of Medicine, University of Debrecen, Debrecen, Hungary; ^6^ Department of Laboratory Medicine, Faculty of Medicine, University of Debrecen, Debrecen, Hungary; ^7^ Hungarian Academy of Sciences-University of Debrecen (MTA-DE) Lendület “Momentum” Hemostasis and Stroke Research Group, Debrecen, Hungary

**Keywords:** antibody-mediated rejection, donor specific antibody, kidney transplantation, thrombin generation, hemostasis

## Abstract

**Introduction:**

The development of *de novo* anti-HLA donor specific antibodies (DSAs) is associated with poor outcomes in kidney transplant recipients. It is surmised that an interaction between DSAs and the graft endothelium cause tissue injury, however, the exact underlying pathomechanism and optimal management of patients with DSAs remain undetermined.

**Aims:**

We hypothesized that in kidney transplant recipients the presence of DSAs induce hemostasis alterations, including hypercoagulability, as assessed by the thrombin generation assay (TGA). Patients and methods. In this observational cohort study, 27 kidney transplant recipients with DSAs (DSA+ group) and 16 without DSAs (DSA– group) were enrolled. Venous blood samples were obtained, and besides routine laboratory tests, von Willebrand factor antigen (VWF), FVIII activity, soluble E selectin (sEsel), soluble P selectin (sPsel), TGA, clot lysis assay (CLA), complement levels (C3, C4) were measured. To correlate results with potential changes in DSA status over time, patients were followed and reassessed 6 ± 1.5 months later.

**Results:**

VWF and sPsel did not differ between groups, but both parameters were increased in the majority of patients. Endogenous thrombin potential (ETP) was significantly higher in the DSA+ group as compared to DSA– patients (median:1666; IQR:1438-2012 vs. 1230; IQR:1097-1659 nM*min, p=0.0019). Follow-up measurements indicated that the observed hemostasis alterations were not transient. CLA parameters, C3 and C4 did not differ between DSA+ and DSA– groups. The extent of anti-HLA II DSA positivity correlated positively with ETP, while tacrolimus levels negatively correlated with ETP and VWF/FVIII levels.

**Conclusions:**

In patients with anti-HLA class II DSAs, thrombin generation was significantly increased as compared to DSA– kidney transplant recipients, suggesting that the presence of antibodies is associated with hypercoagulability. Tacrolimus levels were negatively associated with TGA parameters. Hypercoagulability, associated with the presence of DSAs, may potentially contribute to the pathomechanism of antibody-mediated graft injury, warranting future prospective studies.

## 1 Introduction

Antibody mediated rejection (ABMR) is a common cause of allograft loss in kidney transplant patients (1). The pathophysiology of ABMR is not completely understood as yet, but it is mainly attributed to the action of donor-specific antibodies directed against the human leukocyte antigens (HLA) on the graft endothelium (2). According to available evidence, the presence of anti-HLA donor specific antibodies (DSAs) is associated with a wide-range of potential detrimental effects, including the activation of the complement system, cathepsin-V and galectin-1 expression and secretion in human glomerular endothelial cells, and antibody-dependent cell cytotoxicity on natural killer and myeloid cells, among others (2, 3). Although it is surmised that an interaction between DSAs and the graft endothelium causing tissue injury is mainly responsible for the development of ABMR, the exact underlying pathomechanism and optimal management of patients with DSAs remain undetermined. Moreover, the presence of DSAs does not always translate into rejection, as 30-60% of kidney transplant recipients have antibodies without evidence of rejection (4, 5). Understanding the pathomechanisms resulting in tissue injury may have important clinical relevance, as it could lead to the identification of novel therapeutic targets for the treatment of alloimmune injury, including ABMR.

Surprisingly, although DSAs have been described to cause graft endothelium tissue damage, little is known about potential downstream effects on the hemostasis system. The thrombin generation assay (TGA) is a global hemostasis test, that is suitable for investigating hypo- and hypercoagulability in plasma (6–9). TGA has been described to better mimic the balance of hemostasis occurring *in vivo* as compared to other coagulation procedures (10). In the past years, TGA has been used widely to characterize incompletely understood coagulation mechanisms and to investigate clinical conditions resulting in hypo- or hypercoagulability. In this study, we hypothesized that in kidney transplant recipients, DSAs induce endothelial cell damage and may lead to hypercoagulability, as detected by the TGA, that may ultimately contribute to pathological events causing microvascular obstruction, tissue injury and ABMR.

## 2 Materials and methods

### 2.1 Patients and controls

In this observational case-control study, adult cadaver or living- donor kidney transplant recipients were enrolled at the University of Debrecen, Department of Surgery, Division of Organ Transplantation. Patient enrolment was initiated in September 2019 and was completed in July 2021.

Inclusion criteria of patients were the following: cadaver or living-donor kidney transplant recipient, and age between 18-80 years. Patients with DSAs were classified as cases, while those without antibodies belonged to the control group. Exclusion criteria included acute inflammation, known hemorrhagic or thrombotic diathesis, anticoagulant treatment. As from March 2020, all patients were investigated about the potential acquisition and symptoms of SARS-CoV-2 infection on admission and during follow-up. In case of a suspected infection, patients underwent reverse transcriptase polymerase chain reaction testing of SARS-CoV-2 RNA extracted from nasopharyngeal/oropharyngeal swabs and were excluded from the study in case of positive test result. After enrollment, main demographic and clinical data were recorded (age, sex, body weight, height, date of transplant, medications including the combination of immunosuppressive treatment, major cardiovascular risk factors: active smoking, arterial hypertension, diabetes mellitus, etc.).

### 2.2 Informed consent

Ethic approval was received from the Institutional Ethics Committee, Ethics Committee of the National Medical Research Council (number of the approval: UD RKEB/IKEB H.5228-2019, H.0282-2021). All enrolled patients had been informed about the study and gave written informed consent.

### 2.3 Blood sampling and laboratory measurements

Venous blood was drawn on two occasions from the patients in the study: at baseline and at 6 months post-baseline. Routine laboratory tests including electrolytes, liver and kidney function tests, high-sensitivity C-reactive protein measurement (hsCRP), complete blood count were carried out immediately from the blood samples by standard laboratory methods (Roche Diagnostics, Mannheim, Germany and Sysmex Europe GmbH, Hamburg, Germany). Graft function was evaluated based on serum creatinine and eGFR (ml/min/1.73m^2^). Proteinuria was measured from morning urine specimens (urine protein and urine protein/creatinine ratio). Measurements of tacrolimus trough levels were performed from whole blood using a conventional immunoassay method (target concentration range: 5-15 μg/L). For the examination of hemostasis tests, blood samples were collected to vacutainer tubes containing 0.109 M sodium citrate (Becton Dickinson, Franklin Lane, NJ) and were processed immediately (centrifugation twice at 1500 g, room temperature, 15 min). Screening tests of coagulation (prothrombin time, activated partial thromboplastin time, and thrombin time) and fibrinogen levels according to the method of Clauss were performed immediately on a BCS coagulometer using routine methods (Siemens Healthcare Diagnostic Products, Marburg, Germany). For the execution of soluble markers of endothelial damage, TGA, and the clot lysis assay, aliquots of citrated plasma were stored at -80°C until analysis. To avoid potential bias due to calibration, baseline and follow-up samples were measured within the same run.

### 2.4 DSA measurements

To detect DSAs, the high-sensitivity flow cytometric method of Luminex^®^ Single Antigen Bead assay (One Lambda Inc., Los Angeles, CA) was performed as described previously (11, 12). With the SAB technique, phenotype beads are coated with a single HLA class I (HLA-A, -B, -C) or class II (HLA-DR, -DQ, -DP) recombinant antigen (13). Pre-screening for DSAs were achieved by using pooled antigen panels that provide information on the presence or absence of antibodies belonging to a particular class of HLA. As second step, determination of antibody specificity with highest sensitivity and degree of resolution to the allelic antigen level was achieved by using the SAB technique, providing the results as mean fluorescence intensity (MFI). DSA positivity was defined above the limit value of 500 MFI. The presence of DSAs was screened at baseline and at follow-up in all patients and patients were grouped as DSA+ or DSA– based on the result.

### 2.5 Histopathology

All adequate for-cause (“indication”) renal transplant biopsies performed within one year from baseline were included in the study from consenting patients. The biopsies were subjected to light microscopy with hematoxylin and eosin (HE) staining, periodic acid-Schiff (PAS), and Masson’s trichrome stains, immunofluorescent studies (IgG, IgM, IgA, C3c, fibrinogen, collagen: control staining). Histological lesions were semiquantitatively scored according to the Banff consensus, as reported previously (14). The presence/absence of microvascular obstruction was evaluated by HE staining, and compared with the levels of investigated hemostasis parameters.

### 2.6 Soluble markers of endothelial damage and measurements of complement components (C3, C4)

Von Willebrand factor (VWF) antigen level was measured by an automated latex-particle based immunoassay (Siemens Healthcare Diagnostic Products, Marburg, Germany) as previously described using a Siemens BCS coagulometer (reference interval: 50-160%) (15). Factor VIII activity was assayed by a chromogenic method on a BCS coagulometer according to the manufacturer’s description (reference interval: 60-168%) (15).

Soluble P selectin (sPsel) and soluble E selectin (sEsel) levels were measured using a sandwich enzyme-linked immunosorbent assay (Quantikine ELISA, Bio-Techne, Abingdon, UK) (sEsel reference interval: 13.5-51.3 ng/mL; reference limit of sPsel: 37.9 ng/mL).

Complement levels C3 and C4 were determined by nephelometry (Siemens BNII nephelometer system, Munich, Germany) using reagents from Siemens AG (antisera to human C3c and C4), according to the manufacturer’s description (C3 reference interval: 0.9-1.8 g/L, C4 reference interval: 0.1-0.4 g/L).

### 2.7 Thrombin generation measurements

TGA was performed from stored platelet-free plasma as described previously using the Thrombinoscope CAT (Calibrated Automated Thrombogram, Maastricht, The Netherlands) assay, based on the manufacturer’s instructions (Diagnostica Stago, Asnieres, France) (16). Briefly, 80 μL of plasma was incubated with 20 μL PPP-Reagent™ (containing 5 pM recombinant tissue factor and 4 μM phospholipids) in black round-bottomed 96-well microplates (Greiner Bio, One North America Inc., Monroe, MI) for 10 minutes. For each sample, a calibrator (α2-macroglobulin-thrombin complex with known thrombin activity: Thrombin Calibrator™) was run in parallel, to correct the fluorescence signal for substrate consumption and to compensate plasma color variability. Thrombin generation was initiated by the addition of 20 μL of FluCa-Kit™ (fluorescent substrate and a buffer solution with CaCl_2_: Fluo Buffer). All measurements were performed in duplicates. Fluorescence was detected by a Fluoroskan Ascent^®^ fluorimeter (Thermo Fischer Scientific, Waltham, MA) and the TGA curves were analyzed by the Thrombinoscope software (Thrombinoscope BV, Maastricht, The Netherlands). Thrombin generation curves were characterized by the following parameters (calculated and presented by the Thrombinoscope software): 1/Lag time: the moment from the initiation of the test until thrombin generation starts (the time needed for the first amounts of thrombin to be generated), 2/Endogenous Thrombin Potential (ETP): the area under the curve, 3/Peak thrombin: the highest thrombin concentration formed, 4/Time to peak: the time until the peak thrombin.

### 2.8 Clot lysis measurements

Clot lysis assay (CLA) was conducted based on previously established protocols (17). Briefly, plasma samples were gently thawed in a 37°C water bath. In the wells of a 96-well microtiter plate (Greiner Bio-One International GmbH, Kremsmünster, Austria), a clot induction and lysis mix was prepared in HEPES buffer (10 mM HEPES, 150 mM NaCl, 0,05% Tween20, pH 7.4), where citrated plasma was mixed with 1000-fold diluted human tissue (Innovin, Siemens, Marburg, Germany) and 100 ng/mL recombinant tissue type plasminogen activator (rt-PA, Alteplase, Boehringer Ingelheim, Ingelheim, Germany). The plasma was diluted with buffer 1.2 times. Clotting and subsequent lysis were induced by pipetting HEPES buffer containing CaCl_2_ (21 mM) into each sample well automatically. Turbidity was monitored at 340 nm using a TECAN Infinite m200 microplate reader (TECAN Trading AG, Mannedorf, Switzerland) every minute for 300 min at 37°C. All samples were tested in quadruplicates. The Shiny App software tool (18) was used to analyze curves. Clot formation and lysis were defined using the following variables calculated from the turbidimetric curves: maximum absorbance, time to maximum absorbance, various clot lysis time (CLT) points: 10%CLT, 50%CLT, 90%CLT, and area under the curve (CLA AUC). Clot lysis times were defined as the time from the 10%, 50%, or 90% point, from clear to maximum turbidity, to the 10%, 50%, or 90% point in the transition from maximum turbidity to the final baseline turbidity, respectively.

### 2.9 Statistical analysis

Statistical analysis was performed by the Graphpad Prism 9.0 software (Graphpad Prism Inc, La Jolla, CA). Normality of data was studied using the Shapiro-Wilk test. Normally distributed data is presented as mean ± standard deviation (SD), non-parametric data as median and interquartile range (IQR). For two-group analyses, Student’s t-test or Mann-Whitney U-test were performed, depending on the result of the normality tests. Wilcoxon signed-rank-test was used to analyze paired data sets. Spearman or Pearson’s correlation coefficient were used to determine the strength of correlation between continuous variables. Differences between categorical variables were assessed by χ^2^ test or by Fisher’s exact where appropriate.

## 3 Results

### 3.1 Baseline characteristics in patients

In this study, 43 kidney transplant recipients were included, baseline characteristics of patients are shown in **Table 1**. Sixteen patients without DSAs (DSA– group) and 27 patients with DSAs (DSA+ group) were enrolled. The two groups did not differ significantly regarding baseline demographic data, routine laboratory parameters and the applied immunosuppressive regimen. The mean age was 45.7 ± 10.9 years in the DSA– group and 46.4 ± 12.6 years in the DSA+ group. The mean time after transplantation was 19±12 months in the DSA– group, and 27±19 months in the DSA+ group. In the DSA+ group, anti-HLA class II antibodies were demonstrated in the majority of patients (n=22, 82%). The most frequent cardiovascular risk factor was hypertension in the study cohort. Most patients received a combination of tacrolimus, mycophenolic acid (MPA) and glucocorticoids as immunosuppressive therapy.

**Table 1:** Baseline characteristics of the study population at baseline.

Variables	DSA –	DSA +	*p*
Number of individuals, *n*	16	27	–
Age, year	45.7 ± 10.9	46.4 ± 12.6	0.8612
Male sex, *n* (%)	11 (61)	14 (52)	0.3475
BMI, kg/m^2^	25.9 ± 5.4	27.4 ± 4.9	0.3771
Time post-transplant, months	19 ± 12	27 ± 19	0.2856
Number of transplantations, *n* (%)			
One	12 (66)	14 (52)	0.2860
Two	4 (22)	12 (44)	
Three	0 (0)	1 (4)	
Living donor	1 (6)	2 (7)	>0.9999
DSA HLA specificity, *n* (%)			
Class I	–	4 (15)	–
Class II	–	15 (56)	–
Class I and Class II	–	7 (26)	–
Appearance of DSAs post-transplant, months	–	19 ± 16	–
Cardiovascular risk factors, *n* (%)			
Arterial hypertension	13 (81)	22 (81)	>0.9999
Active smoker	5 (31)	3 (11)	0.1250
Diabetes mellitus	3 (19)	5 (19)	>0.9999
Laboratory parameters (blood)			
Urea, mmol/L	7.4 (6.6-8.9)	8.6 (7.1-12.8)	0.1708
Creatinine, µmol/mL	107 (96-189)	137 (109-163)	0.3148
eGFR, mL/min	59 ± 24	51 ± 20	0.2439
Total protein, g/L	70 (67-77)	69 (65-73)	0.5255
Albumin, g/L	46 ± 4	44 ± 5	0.1019
hsCRP, mg/L	1.9 (0.9-3.5)	3.4 (1.5-5.8)	0.1667
WBC, G/L	7.9 ± 2.4	7.5 ± 2.0	0.5643
Hemoglobin, g/L	130 (124-160)	129 (119-134)	0.1910
Platelet count, G/L	222 (172-271)	224 (179-266)	0.5890
PT, s	8.5 (7.9-9.0)	8.2 (7.8-8.6)	0.1968
APTT, s	30.1 ± 4.4	28.2 ± 3.3	0.1227
Laboratory parameters (urine)			
Protein, mg/L	40 (39-106)	51 (40-203)	0.1417
Protein/creatinine ratio, mg/mmol	19 (10-99)	17 (11-65)	0.7489
Immunosuppressive therapy, *n* (%)			
Tacrolimus+MPA+Glucocorticoid	13 (82)	17 (63)	0.2069
Tacrolimus+MPA	0 (0)	2 (7)	0.5216
mTORi+Tacrolimus+Glucocorticoid	0 (0)	2 (7)	0.5216
mTORi+Tacrolimus	1 (6)	1 (4)	>0.9999
mTORi+MPA+Glucocorticoid	0 (0)	2 (7)	0.5216
mTORi+MPA	0 (0)	1 (4)	>0.9999
Tacrolimus+MPA +mTORi+Glucocorticoid	1 (6)	1 (4)	>0.9999
Tacrolimus+Glucocorticoid	1 (6)	1 (4)	>0.9999
Tacrolimus levels, μg/L	7.7 ± 2.4	7.2 ± 2.6	0.5117

Continuous variables are expressed as mean ± standard deviation or median (interquartile range). Categorical variables are indicated as number (percentage). APTT, activated partial thromboplastin time; BMI, body mass index; DSA, donor specific antibodies; eGFR, estimated glomerular filtration rate; HLA, human leukocyte antigen; hsCRP, high sensitivity C-reactive protein measurement; MPA, mycophenolic acid; mTORi, mammalian target of rapamycin inhibitors; n, number; PT, prothrombin time; WBC, white blood cell.

### 3.2 Soluble markers of endothelial damage, TGA and clot lysis assay results at baseline

**Figure 1:**
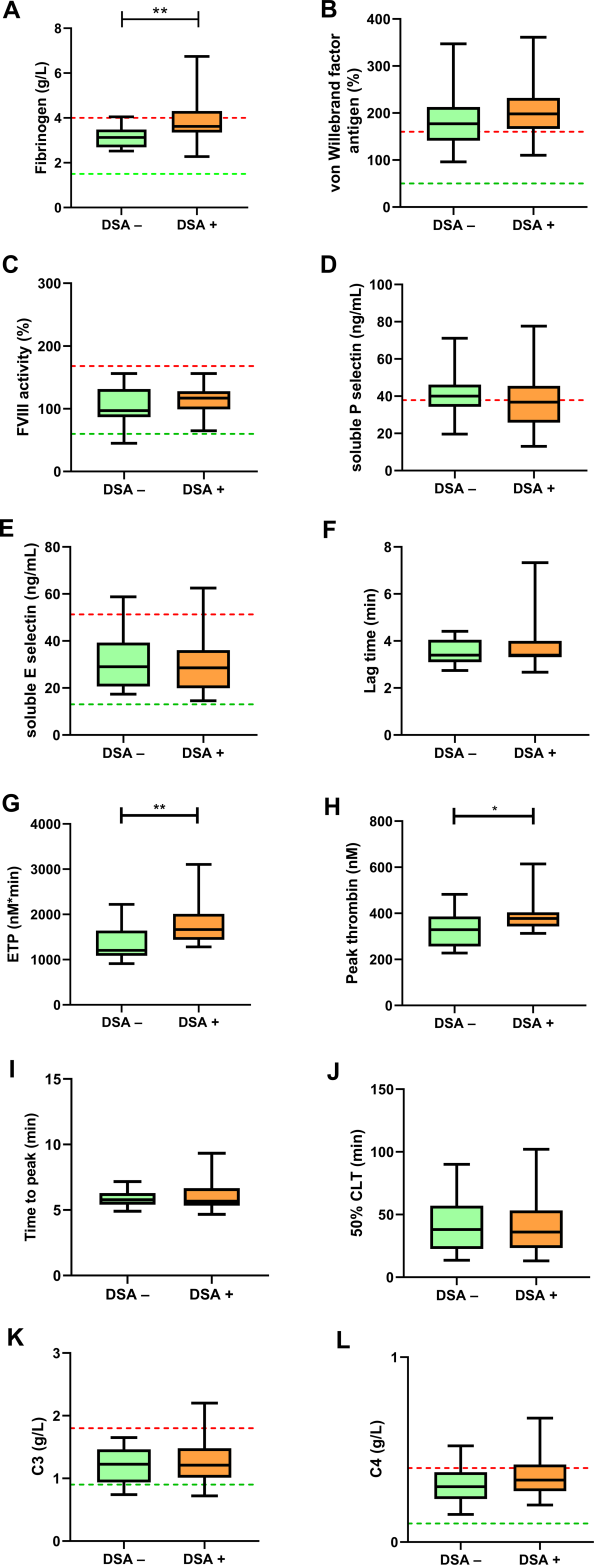
Soluble markers of endothelial damage, thrombin generation assay (TGA) and clot lysis assay (CLA) results at baseline. Fibrinogen **(A)**, von Willebrand factor antigen **(B)**, factor VIII activity **(C)**, soluble P selectin **(D)**, soluble E selectin **(E)**, TGA parameters including lag time **(F)**, ETP **(G)**, peak thrombin **(H)**, time to peak **(I)**, 50% clot lysis time (CLT) of the CLA **(J)**, complement component C3 **(K)** and C4 **(L)** are shown in kidney transplant recipient patients without (DSA–, green boxes) or with (DSA+, orange boxes) donor specific antibodies at baseline. The lower and upper box boundaries represent the 25^th^ and 75^th^ percentiles, respectively, horizontal solid lines represent the median, and whiskers indicate the range. The lower (green) and upper (red) levels of reference intervals as provided by the manufacturer are indicated with dotted lines (reference intervals are not available for TGA and CLA). CLT, clot lysis time; DSA; donor specific antibody; ETP, endogenous thrombin potential; FVIII, factor VIII. *p<0.05, **p<0.01.

At baseline, fibrinogen levels were significantly higher in the DSA+ group of patients as compared with the DSA– group (median: 3.6; IQR: 3.4-4.3 vs. 3.1; IQR: 2.7-3.5 g/L, p=0.0081) (**Figure 1A**). VWF antigen and sPsel levels were above the upper limit of reference at baseline in a large fraction of patients, but no difference was observed between groups (**Figures 1B, D**). VWF antigen and sPsel levels showed a fair correlation (Spearman r= 0.3788, 95%CI: 0.0794 to 0.6155, p=0.0122) in the studied cohort. FVIII activity and sEsel did not differ between groups (**Figures 1C, E**). Among the baseline TGA parameters, ETP was significantly higher in the DSA+ group as compared to DSA– patients (median:1666; IQR: 1438-2012 vs. 1230; IQR: 1097-1659 nM*min, p=0.0019) (**Figure 1G**). Peak thrombin was also significantly higher in patients with DSAs vs. those without (median: 377.4; IQR: 342.9-404.5 vs. 328.9; IQR: 256.5-386.3 nM, p=0.0203), while other TGA parameters were not significantly different between groups (**Figures 1F, H, I**). CLA parameters, including 50%CLT, as well as C3 and C4 levels did not differ between DSA+ and DSA– groups at baseline (**Figures 1J–L**). The prevalence of C3 hypocomplementemia did not differ according to the presence of DSAs (DSA+ group: 4/27, 15% vs. DSA– group: 3/16, 19%, p=0.999). Graft type (living donor vs. cadaver) and post-transplant time to DSA presentation did not have an effect on the tested parameters (data not shown).

### 3.3 Associations between TGA parameters, DSA positivity and tacrolimus levels at baseline

**Figure 2:**
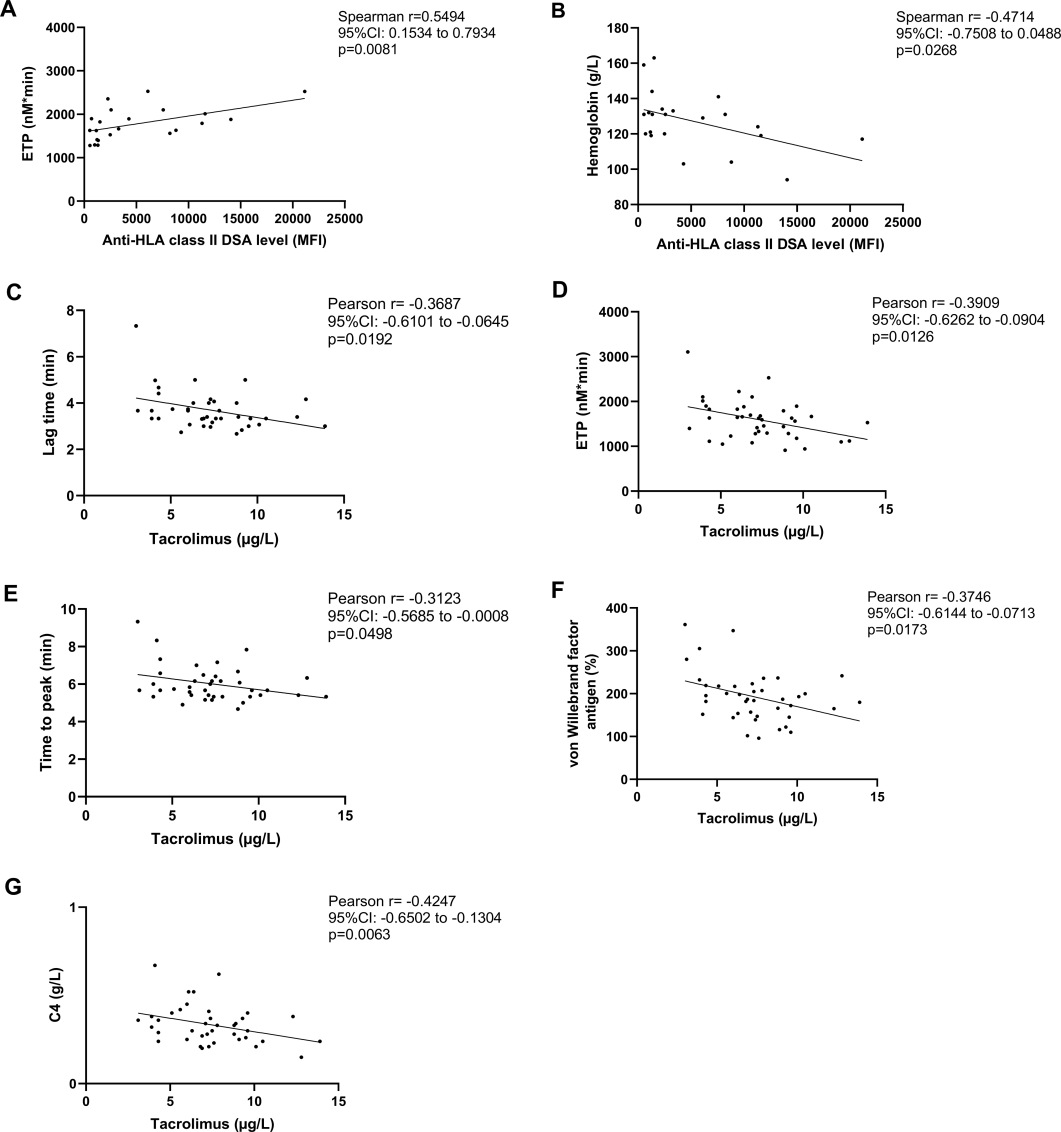
Association between thrombin generation assay (TGA) parameters, donor specific antibody (DSA) positivity and tacrolimus levels at baseline. Correlation of anti-HLA class II DSA levels with endogenous thrombin potential (ETP) **(A)** and hemoglobin concentration **(B)** in the investigated cohort of kidney transplant recipient patients at baseline. Correlation between tacrolimus trough concentrations in the total cohort vs. lag time **(C)**, ETP **(D)**, time to peak **(E)**, von Willebrand factor antigen levels **(F)**, complement component C4 **(G)**. CI, confidence interval; DSA, donor specific antibody; ETP, endogenous thrombin potential; HLA, human leukocyte antigen; MFI, mean fluorescence intensity.

Among the baseline laboratory and hemostasis parameters, ETP showed a significant positive correlation with the extent of anti-HLA class II DSA levels (expressed as MFI, as measured with the Luminex bead array, **Figure 2A**). At the same time, hemoglobin concentration showed a significant negative correlation with DSA MFI (**Figure 2B**). Parameters of the TGA (lag time, ETP, time to peak) showed significant negative correlation with tacrolimus levels (**Figures 2C–E**) in the studied cohort. Similarly, VWF and C4 levels negatively correlated with tacrolimus levels (**Figures 2F, G**).

### 3.4 Soluble markers of endothelial damage, TGA and clot lysis assay results at follow-up

Six months after the baseline examinations, 3 DSA– patients were detected with *de novo* DSAs, while in case of 5 DSA+ patients, the MFI values of anti-HLA antibodies diminished below threshold. One patient was lost to follow-up due to participation in an interventional study. In the follow-up cohort, therefore, 18 patients without DSAs (DSA– group) and 24 patients with DSAs (DSA+ group) were studied (**Table 2**). BMI was found to be significantly higher in the DSA+ group as compared with DSA– patients. Post-transplant time was significantly longer in patients with DSAs. Albumin level was significantly lower while CRP was significantly higher in the DSA+ group vs. those without anti-HLA DSAs.

**Table 2:** Baseline characteristics of patients at 6 months after baseline.

Variables	DSA –	DSA +	*p*
Number of individuals, *n*	18	24	–
Age, year	46.4 ± 12.7	46.7 ± 11.7	0.9386
Male sex, *n* (%)	12 (66)	13 (54)	0.5302
BMI, kg/m^2^	25.1 ± 4.2	28.6 ± 5.5	0.0302
Time post-transplant, months	23.9 ± 11.7	34.6 ± 16.9	0.0481
Number of transplantations, *n* (%)			
One	13 (72)	14 (58)	0.5091
Two	5 (27)	9 (38)	
Three	0 (0)	1 (4)	
DSA HLA specificity, *n* (%)			
Class I	–	5 (21)	–
Class II	–	13 (54)	–
Class I and Class II	–	6 (25)	–
Appearance of DSAs post-transplant, months	–	24 ± 18	–
Cardiovascular risk factors, *n* (%)			
Arterial hypertension	14 (78)	21 (88)	0.4376
Active smoker	5 (28)	3 (13)	0.2562
Diabetes mellitus	2 (11)	6 (25)	0.4307
Laboratory parameters (blood)			
Urea, mmol/L	7.7 (5.3-12.3)	9.4 (7.3-12.1)	0.4307
Creatinine, µmol/mL	117 (98-204)	131 (112-151)	0.7895
eGFR, mL/min	54 ± 24	50 ± 18	0.5547
Total protein, g/L	70 (66-73)	70 (66-76)	0.4216
Albumin, g/L	47 ± 3	44 ± 4	0.0237
hsCRP, mg/L	1.4 (0.6-2.2)	3.1 (0.9-7.7)	0.0266
WBC, G/L	7.6 ± 2.4	7.2 ± 1.8	0.4941
Hemoglobin, g/L	140 ± 17	131 ± 18	0.1170
Platelet count, G/L	208 (174-263)	214 (175-272)	0.7471
PT, s	8.5 (8.0-8.9)	8.3 (8.0-8.8)	0.7309
APTT, s	27.0 (26.0-29.4)	27.1 (25.3-30.1)	0.6748
Laboratory parameters (urine)			
Protein, mg/L	49 (40-116)	56 (40-126)	0.5116
Protein/creatinine ratio, mg/mmol	11 (7-18)	19 (9-42)	0.0827
Immunosuppressive therapy, *n* (%)			
Tacrolimus+MPA+Glucocorticoid	15 (83)	17 (71)	0.3466
Tacrolimus+MPA	0 (0)	2 (8)	0.4983
mTORi +Tacrolimus+Glucocorticoid	0 (0)	2 (8)	0.4983
mTORi+Tacrolimus	2 (11)	0 (0)	0.1777
mTORi+MPA+Glucocorticoid	1 (6)	2 (8)	>0.9999
mTORi+MPA	0 (0)	1 (5)	0.4286
Tacrolimus+MPA +mTORi+Glucocorticoid	0 (0)	0 (0)	–
Tacrolimus+Glucocorticoid	0 (0)	0 (0)	–
Tacrolimus levels, μg/L	6.2 (4.9-7.8)	6.2 (5.6-7.4)	0.9883

Continuous variables are expressed as mean ± standard deviation or median (interquartile range). Categorical variables are indicated as number (percentage). APTT, activated partial thromboplastin time; BMI, body mass index; DSA, donor specific antibodies; eGFR, estimated glomerular filtration rate; HLA, human leukocyte antigen; hsCRP, high sensitivity C-reactive protein measurement; MPA, mycophenolic acid; mTORi, mammalian target of rapamycin inhibitors; n, number; PT, prothrombin time; WBC, white blood cell.

**Figure 3:**
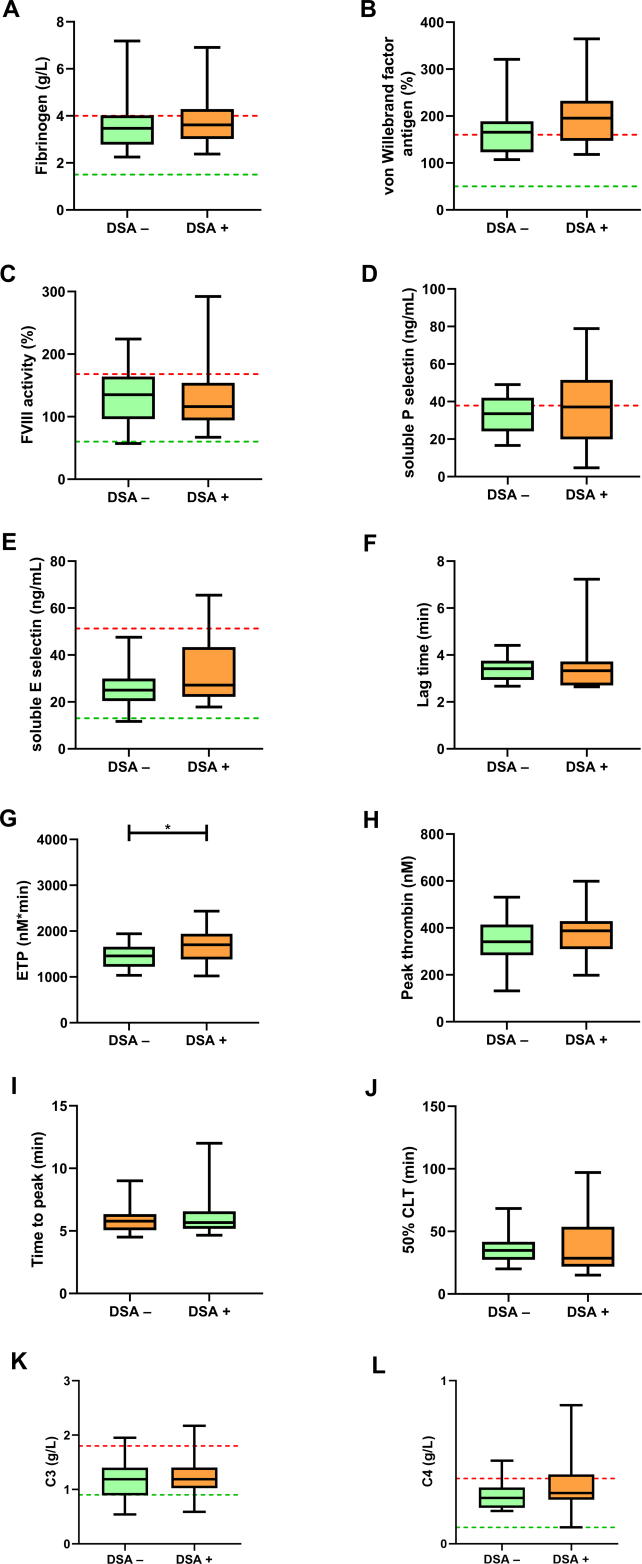
Soluble markers of endothelial damage, thrombin generation assay (TGA) and clot lysis assay (CLA) results at follow-up. Fibrinogen **(A)**, von Willebrand factor antigen **(B)**, factor VIII activity **(C)**, soluble P selectin **(D)**, soluble E selectin **(E)**, TGA parameters including lag time **(F)**, ETP **(G)**, peak thrombin **(H)**, time to peak **(I)**, 50% clot lysis time (CLT) of the CLA **(J)**, complement component C3 **(K)** and C4 **(L)** are shown in kidney transplant recipient patients without (DSA–, green boxes) or with (DSA+, orange boxes) donor specific antibodies at baseline. The lower and upper box boundaries represent the 25^th^ and 75^th^ percentiles, respectively, horizontal solid lines represent the median, and whiskers indicate the range. The lower (green) and upper (red) levels of reference intervals as provided by the manufacturer are indicated with dotted lines (reference intervals are not available for TGA and CLA). CLT, clot lysis time; DSA; donor specific antibody; ETP, endogenous thrombin potential; FVIII, factor VIII. *p<0.05.

At follow up, fibrinogen, VWF antigen levels, FVIII activity, sPsel and sEsel did not differ between groups (**Figures 3A–E**). Consistently with baseline measurements, VWF levels were above the upper limit of reference interval in the majority of patients (**Figure 3B**). Again, ETP was significantly increased in the DSA+ group as compared to the DSA– group (median: 1701; IQR: 1382-1940 vs. 1458; 1221-1660 nM*min, p=0.0381) (**Figure 3G**). At follow up, peak thrombin and time parameters of TGA (lag time and time to peak) showed no difference between groups (**Figures 3F, H, I**). Similarly to baseline, CLA parameters, including 50%CLT, as well as C3 and C4 levels did not differ between DSA+ and DSA– groups (**Figures 3J–L**). The prevalence of C3 hypocomplementemia remained the same at follow-up in both groups.

Renal graft function (e.g. serum creatinine levels, eGFR, proteinuria, urine protein/creatinine ratio) was not significantly different between the investigated groups within the study period (**Tables 1**, **2**). Interestingly, in DSA+ patients, follow-up serum creatinine showed a significant positive correlation with ETP (r=0.5085, 95% CI: 0.1419 to 0.7525, p=0.0094) and peak thrombin (r=0.4263, 95% CI: 0.0374 to 0.7030, p=0.0336), that was not observed in the group of DSA– patients at follow-up (**Supplementary Tables 1**–**4**).

**Figure 4:**
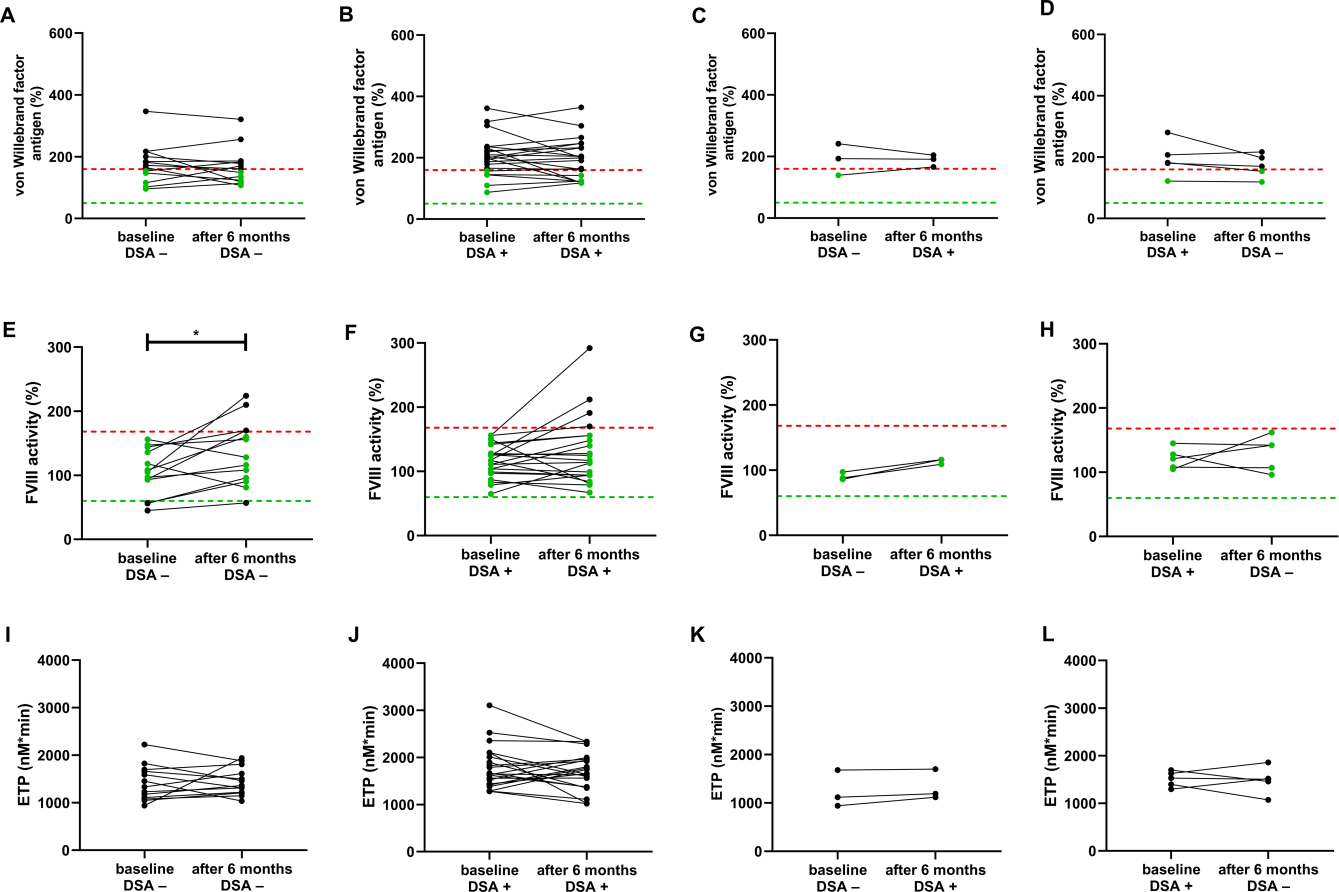
von Willebrand factor antigen, FVIII activity and endogenous thrombin potential (ETP) in different subgroups of patients according to DSA positivity at baseline and at follow-up. von Willebrand factor **(A–D)**, FVIII **(E–H)** and ETP **(I–L)** are shown at baseline and follow-up (after 6 months) in different subgroups of kidney transplant recipient patients according to their DSA positivity at baseline and at follow-up. The lower (green) and upper (red) levels of reference intervals as provided by the manufacturer are indicated with dotted lines (reference interval is not available for ETP). Green dots indicate results within the reference range. DSA; donor specific antibody; ETP, endogenous thrombin potential; FVIII, factor VIII. *p<0.05.

In the subgroups with *de novo* appearance of DSAs or diminishing DSA positivity, VWF antigen levels showed non-significant alterations as compared to baseline (**Figure 4**). It must be noted, however, that in patients with consistent DSA– reports, 43% of patients demonstrated VWF antigen levels below the upper threshold (**Figure 4A**), while only 29% of DSA+ patients had VWF antigen levels within the reference range (**Figure 4B**). In all 3 patients with *de novo* DSA positivity, VWF antigen levels were above the upper limit of reference at follow-up (**Figure 4C**), while no marked differences of VWF antigen levels were observed in patients with diminishing DSAs (**Figure 4D**). Interestingly, FVIII significantly increased in DSA– patients at follow-up (p=0.0200), and a similar, non-significant trend was observed in DSA+ patients (p=0.0938) (**Figures 4E, F**). In both groups, FVIII levels increased above the upper limit of reference in case of 4 patients at follow-up. In all 3 patients with *de novo* DSA positivity, FVIII levels increased at follow-up (**Figure 4G**), while in patients with diminishing DSAs variable changes occurred in FVIII levels (**Figure 4H**). ETP showed non-significant alterations as compared to baseline in all investigated subgroups (**Figures 4I–L**). Peak thrombin showed parallel results to ETP in all subgroups, without significant alterations at follow-up (data not shown).

### 3.5 Associations between TGA parameters, DSA positivity and tacrolimus levels at follow-up

**Figure 5:**
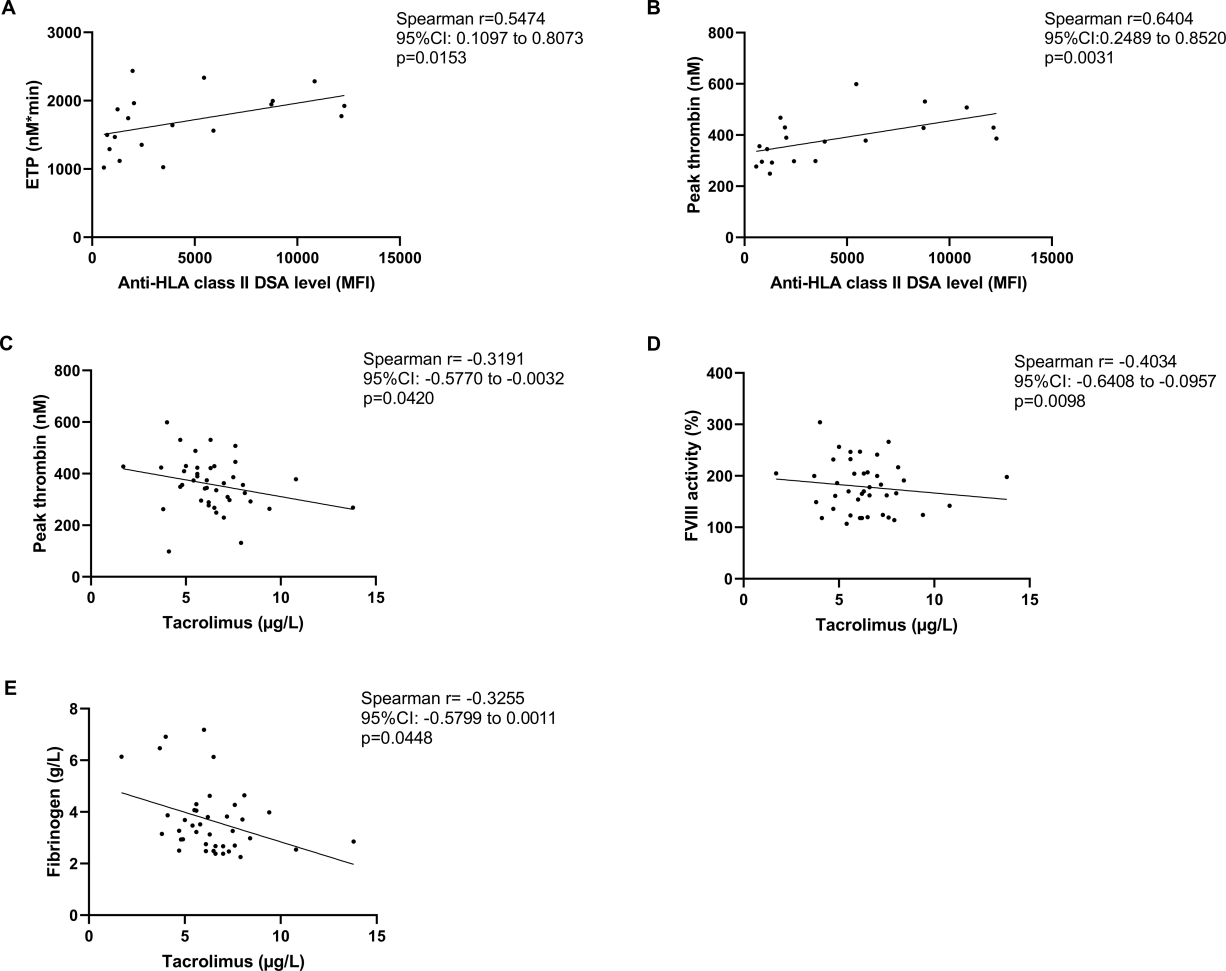
Association between thrombin generation assay (TGA) parameters, donor specific antibody (DSA) positivity and tacrolimus levels at follow-up. Correlation of anti-HLA class II DSA levels with endogenous thrombin potential (ETP) **(A)** and peak thrombin concentration **(B)** in the investigated cohort of kidney transplant recipient patients at follow-up (6 months after baseline). Correlation between tacrolimus trough concentrations in the total cohort vs. peak thrombin **(C)**, FVIII activity **(D)**, and fibrinogen levels **(E)** at follow-up. CI, confidence interval; DSA, donor specific antibody; ETP, endogenous thrombin potential; FVIII, factor VIII; HLA, human leukocyte antigen; MFI, mean fluorescence intensity.

Consistently with baseline results, at 6 months the extent of thrombin generation showed a significant positive correlation with DSA positivity (**Figures 5A, B**). Peak thrombin, as well as FVIII levels and fibrinogen showed significant negative correlation with tacrolimus levels in the studied cohort at follow-up (**Figures 5C–E**). The extent of anti-HLA class II DSA positivity did not correlate with tacrolimus levels at any time during the investigations (data not shown). Post-transplant elapsed time did not show an association with any of the measured hemostasis parameters (data not shown). VWF antigen and FVIII levels showed significant positive correlation at baseline and at follow-up (Spearman r=0.5876, 95%CI: 0.3408 to 0.7587, p<0.0001 and r=0.5919, 95%CI: 0.3429 to 0.7631, p<0.0001), without relevant differences among the investigated subgroups.

### 3.6 Associations between TGA parameters, microvascular changes in follow-up renal graft biopsies and various laboratory parameters

**Figure 6:**
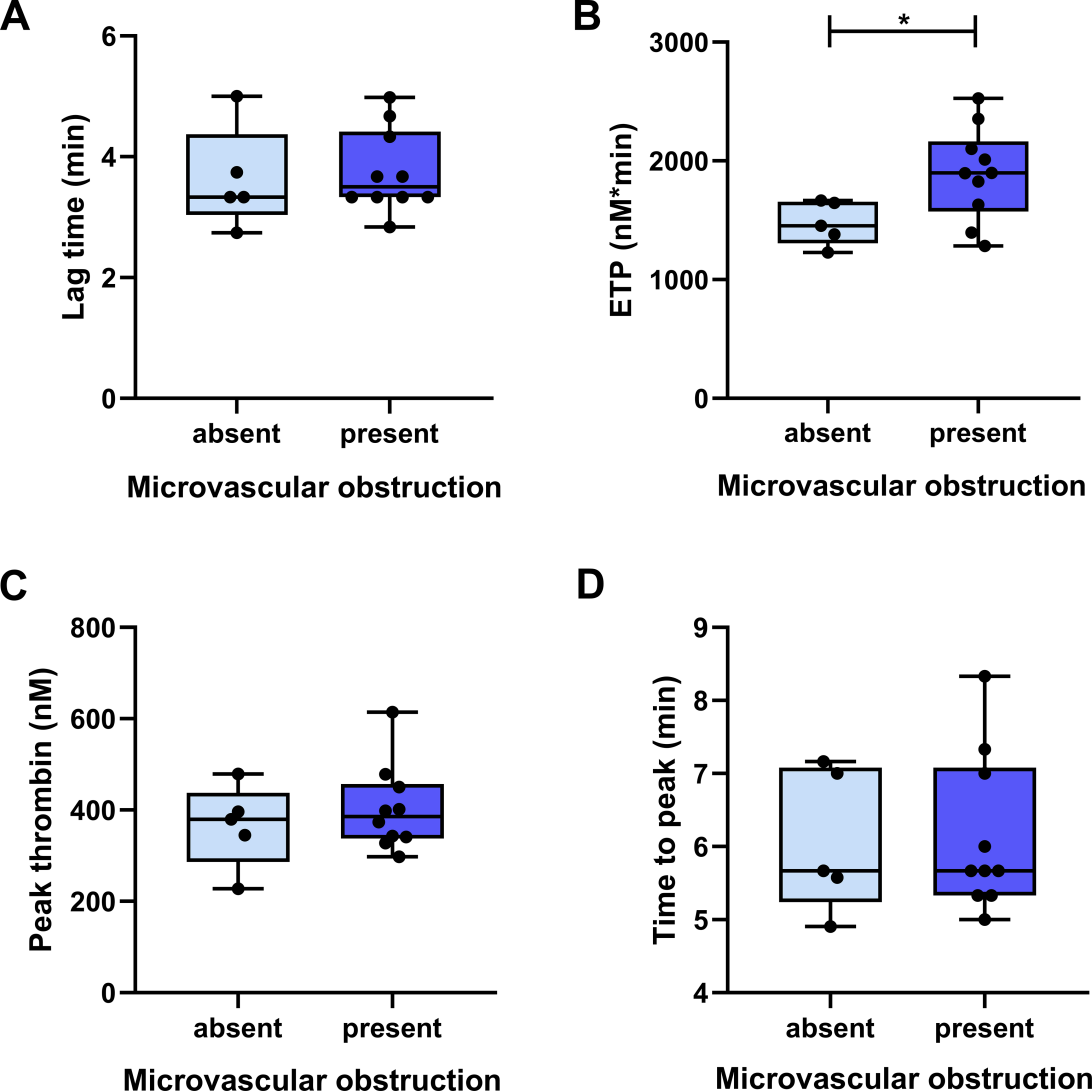
Association between thrombin generation assay (TGA) parameters and the presence of microvascular obstruction in follow-up graft biopsies. Panels **(A–D)** show TGA parameters according to the presence (light blue boxes) or absence (dark blue boxes) of microvascular obstruction in follow-up graft biopsies (within 1 year from baseline), as determined by light microscopy and hematoxylin and eosin staining. The lower and upper box boundaries represent the 25^th^ and 75^th^ percentiles, respectively, horizontal solid lines represent the median, and whiskers indicate the range. The number of patients in each group is indicated with dots (microvascular obstruction absent: n=5 vs. present: n=10). ETP, endogenous thrombin potential, *p<0.05.

Fifteen renal graft biopsies were performed within one year from baseline in consenting patients (n=2 in DSA– patients, n=13 in DSA+ patients). Histological lesions, scored according to the Banff consensus are summarized in **Supplementary Table 5**. In patients demonstrating renal graft microvascular obstruction (n=10 cases, all DSA+ patients), admission ETP was significantly higher as compared to those without microvascular changes (n=5 cases) (**Figure 6**, ETP median: 1898, IQR: 1573-2165 vs. 1453, IQR: 1306-1656 nM*min, p=0.0425). The presence of microvascular obstruction was not associated with the levels of other hemostasis or complement parameters tested in this cohort (data not shown). In DSA– patients, the extent of thrombin generation (as demonstrated by ETP or peak thrombin) showed positive correlation with C3 and C4 levels (**Supplementary Tables 6**, **7**), while in DSA+ patients ETP/peak thrombin was significantly associated with CRP, fibrinogen, VWF levels and FVIII activity, suggesting stronger link between inflammation and thrombin generation in this subgroup (**Supplementary Tables 8**, **9**). Of note, sPsel did not demonstrate a significant correlation with any of the tested TGA parameters, at baseline or follow-up, indicating a lack of association between platelet hyperreactivity and increased thrombin generation in the tested cohort, regardless of the presence of DSAs.

## 4 Discussion

ABMR remains a leading immunological cause of allograft loss after renal transplantation (1). Although the exact underlying mechanisms of ABMR are still not entirely clear, DSA formation is the main pathologic event associated with allograft failure (19). It is conceivable that the hemostasis system could play a significant role in several pathological processes responsible for graft function loss, based on the several intersection points between coagulation, fibrinolysis and immunity. Interestingly, only a handful of papers have been published on selected hemostasis markers in patients with kidney transplants, and according to our knowledge, TGA has not been carried out as yet in such patient cohort (20–23).

**Figure 7:**
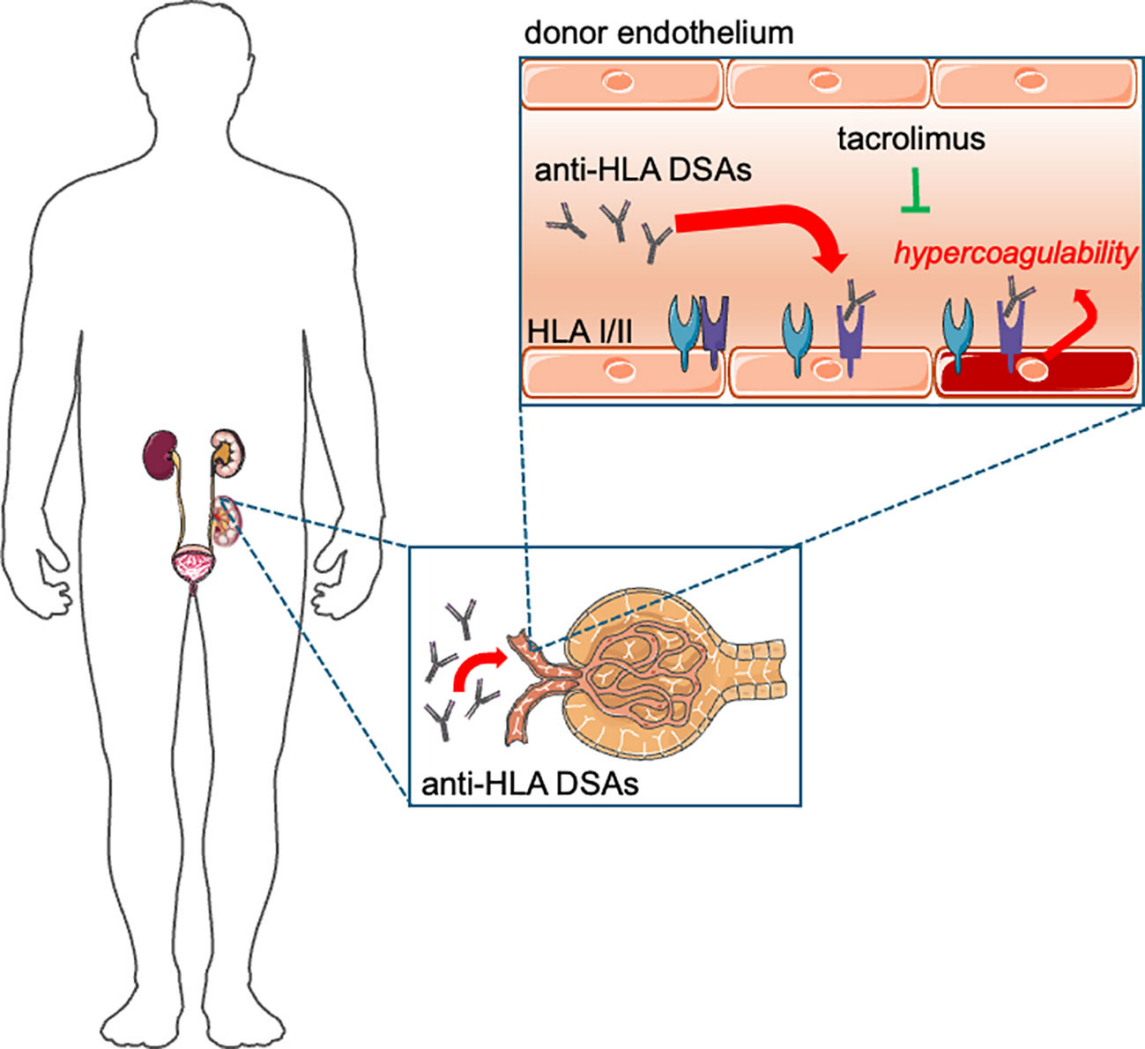
A scheme illustrating the effect of anti-HLA donor specific antibodies (DSAs) on thrombin generation and the effect of tacrolimus. In kidney transplant recipients with anti-HLA class II DSAs, thrombin generation was significantly increased as compared to those without DSAs, suggesting that the presence of antibodies is associated with hypercoagulability. Tacrolimus levels were negatively associated with the extent of thrombin generation.

As to our knowledge, here we show for the first time that thrombin generation is enhanced in kidney transplant patients with DSAs (the summary of our findings is illustrated in **Figure 7**). In the DSA+ group of patients, ETP was significantly elevated as compared to the DSA– group at baseline and at 6 months later, indicating a permanent hypercoagulable state in patients with DSAs. Moreover, the extent of anti-HLA class II DSA positivity showed a significant positive correlation with ETP at both occasions, indicating a direct association between these type of DSAs inducing a prothrombotic condition. Although the immunologic risk for humoral rejection has been clearly defined by multiple studies in patients with anti-HLA class II DSAs, the exact pathomechanism has not been clarified, as yet (24, 25). Our results indicate that the presence of these antibodies are associated with a prothrombotic condition and hypercoagulation, and higher DSA levels indicate a highly increased prothrombotic state, which may have potential implications for the clinical practice. As a potential causal association between increased thrombin generation leading to ABMR and graft rejection, a significantly increased ETP was found in patients with microvascular obstruction in follow-up graft biopsies as compared to those without microvascular changes. In this study, the number of patients with *de novo* DSAs was low (n=3) to draw firm conclusions, however, it must be noted that ETP showed a slight increase after the appearance of DSAs, which might also indicate a direct effect. Moreover, evolution of renal graft function (serum creatinine, eGFR at 6 months follow-up) demonstrated a significant correlation with ETP and peak thrombin in DSA+ patients, that was not observed in the DSA– patient group. Prospective studies with larger patient cohorts are needed to confirm these initial observations. For the first time, we provide data that tacrolimus levels negatively correlate with TGA parameters. Interestingly, such direct association between tacrolimus levels and anti-HLA class II DSAs was not detected. These results suggest that TGA values may strongly depend on the administered immunosuppression regimen, moreover, an indirect link between inflammation and thrombin generation is suspected, indicating that the extent of immunosuppression may be associated with a pathomechanism leading to hypercoagulation. A significant negative correlation between acute phase proteins, i.e. C4, FVIII/VWF and tacrolimus levels also supports this hypothesis. Recently, it has been shown in elegant studies, that blood pro-inflammatory cytokines are increased in kidney transplant patients with DSAs and associated with worse graft survival even in the absence of histology rejection (26). Notably, according to our findings, the clot lysis assay was not different across the investigated subgroups, suggesting that only the balance of coagulation and not that of fibrinolysis is distorted in kidney transplant patients with DSAs. These observations may indicate a so-far unexplored role of hypercoagulation and prothrombotic hemostasis balance in the pathomechanism of ABMR that warrant further studies. The significance of these results may be dual: firstly, TGA may help to determine the timing of kidney biopsy and the need to modify the immunosuppression medications. Secondly, a potential role of hypercoagulation in the pathomechanism of humoral rejection implies that prophylactic anticoagulation might be beneficial for patients with hypercoagulability. It must be noted, however, that our study does not specifically address whether hypercoagulation exists in DSA+ cases where the transplanted organ is not attacked by humoral immunity, a condition known as accommodation (27). As our study was a hypothesis-generating study and was not designed to answer these specific questions, future investigations are required to assess the clinical impact of the results. Based on our study, TGA might be a useful method to monitor hypercoagulability in patients with DSAs, however, more prospective studies are needed to confirm an association between increased thrombin generation and hypercoagulability leading to graft rejection.

It must be pointed out, that during the follow-up of 6 months, results on TGA, as well as VWF, sEsel and sPsel levels did not essentially change in the investigated subgroups with or without DSAs, indicating that the observed hemostasis alterations were not transient in these patients. Although we suspected a significant difference between VWF levels of patients with or without DSAs, such association was not confirmed in this cohort. VWF antigen levels were increased above the upper limit of reference in the majority of investigated patients, unrelated to the presence of DSAs. Notably, it has been previously reported that *in vitro* anti-HLA antibodies promote VWF secretion that may lead to vascular inflammation and microthrombosis (28), while in the same report, similarly to our findings, tacrolimus treatment was associated with decreased VWF levels in the sera of kidney recipients with or without DSAs. Therefore, previously published results are in line with our findings indicating that VWF levels are likely to be regulated by inflammation/immunosuppression in kidney transplant patients. Nevertheless, the relatively high ratio of patients in our cohort with elevated VWF levels despite the absence of DSAs warrants further research. Increased VWF is a promising surrogate marker of renal vessel damage in the transplanted kidney (29, 30), but the source of increased VWF has not been clearly identified. Interestingly, sEsel, indicative of endothelial damage, was not increased in our cohort, while sPsel, a marker of endothelial damage and platelet activation, was increased in the majority of patients with or without DSAs. This may be explained by the fact that increased VWF levels might not just directly reflect the actual degree of endothelial damage, but it may reflect platelet activation, similarly to sPsel (31, 32). The fair but significant correlation between sPsel and VWF levels at baseline and between FVIII and VWF levels in this cohort also suggest that increased VWF levels might originate, at least in part, from platelets and elevated levels may not be solely attributable to endothelial damage (31, 33). On the other hand, a lack of association between sPsel and TGA parameters indicates that platelet hyperreactivity does not contribute to increased thrombin generation in the tested cohort, regardless of the presence of DSAs. As VWF is an acute phase protein, increased levels may not directly reflect endothelial dysfunction (32). Nevertheless, it has been suggested that the measurement of VWF levels may be more useful in this respect than other acute phase reactants or inflammatory markers (e.g. CRP) as elevated VWF levels may imply vascular disease (32).

Despite the critical role of DSAs in allo-immune graft rejection, early identification of the process of ABMR remains a challenge. In search of biomarkers and therapeutic targets for acute rejection, strategies targeting the hemostasis system may be beneficial to prevent the development of microthrombosis, ischemia and diminished microcirculation during ABMR. Underlying mechanisms of microvascular obstruction may involve the activation of both coagulation and complement systems as a response to DSAs (34). Several studies have investigated the impact of circulating complement-activating DSAs on organ transplant outcomes (35). The complement system is paramount in the clearance of pathogens, yet it is increasingly recognized as a potential key pathway leading to allograft injury (36). *In vitro* studies have suggested that various coagulation serine proteases, including thrombin are able to directly activate complement, for instance C3, however, the *in vivo* relevance of coagulation proteases bypassing complement-mediated activation mechanisms is still limited (37). To test the possibility whether a thrombo-inflammatory cross-talk between the complement system and coagulation is present in the studied patient cohort, the levels of C3 and C4 were assessed. C3 and C4 levels and the prevalence of C3 hypocomplementemia were not different between DSA+ and DSA– patients at admission or at follow-up. Interestingly, significant positive associations were found between C3 and C4 levels and TGA parameters, regardless of the presence/absence of DSAs. Similarly to other acute phase proteins tested in this cohort, C4 levels demonstrated a significant negative correlation with tacrolimus levels. These results suggest that the above described hemostasis alterations, including increased thrombin generation and hypercoagulation might not be necessarily accompanied with the activation of C3. In the light of potential novel therapeutic options to avoid organ rejection, including increasing number of complement therapeutics, future studies are warranted to define key pathomechanisms, including the role of thromboinflammation and the relative importance of particular pathways associated with graft failure under given clinical conditions (38).

## 5 Conclusions

In summary, our findings demonstrate for the first time that the TGA may have useful applications in the evaluation of hemostasis balance in kidney transplant patients with DSAs. The extent of thrombin generation was significantly increased in patients with DSAs as compared to those without. The extent of anti-HLA class II DSA positivity positively correlated with ETP at baseline and after 6 months as well, indicating that this observation was not transient. Tacrolimus levels negatively correlated with thrombin generation parameters, and with VWF antigen/FVIII levels. VWF antigen levels did not show an association with DSA positivity in this cohort, indicating an independent pathomechanism for the observed increase in the majority of patients. On the other hand, hypercoagulability, associated with the presence of DSAs, may potentially contribute to the pathomechanism of antibody-mediated graft injury, as microvascular obstruction in follow-up graft biopsies were accompanied by significantly higher ETP as compared to those without microvascular changes. More studies are needed to assess the importance of hypercoagulability in graft rejection and the potential use of TGA in monitoring this complication.

## 6 Limitations

Results of this study should be interpreted in the context of its limitations and strengths. The study was single-centered, which contributed to the limited sample size, but it had the advantages of uniform sample handling and patient care. Unfortunately, patient enrollment had to stop after the second wave of COVID-19 in Hungary, as it became impossible to enroll unaffected patients (without COVID-19 infection/post-COVID-19 status or post-vaccination status), all of which may have potentially influenced the balance of hemostasis, TGA results and follow-up data. Given the low number of patients with *de novo* DSAs during the follow-up, conclusions derived from these observations must be verified in larger prospective cohorts. The effect of autoantibodies other than anti-HLA DSAs (anti-endothelial cell antibodies, anti-major histocompatibility complex I-related chain A: anti-MICA) were not investigated in this study. The limited sample size hampered the ability to draw firm conclusions on the relationship between hypercoagulation and the evolution of the renal graft function and the occurrence of ABMR over time. As the study was a hypothesis generating study, it was not designed to find out whether TGA or other hemostasis assays might provide help in the personalized management of patients with ABMR or affect the histopathological findings directly related to graft rejection.

## Data Availability

The raw data supporting the conclusions of this article will be made available by the authors, upon reasonable request. Further inquiries can be directed to the corresponding author.

## References

[1] Allison SJ. Transplantation: The molecular landscape of ABMR. Nat Rev Nephrol. (2015) 11(5):255. doi: 10.1038/nrneph.2015.41 25825080

[2] Zhang R. Donor-specific antibodies in kidney transplant recipients. Clin J Am Soc Nephrol. (2018) 13:182–92. doi: 10.2215/CJN.00700117 PMC5753302 28446536

[3] Clotet-Freixas S McEvoy CM Batruch I Pastrello C Kotlyar M Van JAD. Extracellular matrix injury of kidney allografts in antibody-mediated rejection: A proteomics study. J Am Soc Nephrol. (2020) 31:2705–24. doi: 10.1681/ASN.2020030286 PMC7608967 32900843

[4] Oweira H Ramouz A Ghamarnejad O Khajeh E Ali-Hasan-Al-Saegh S Nikbakhsh R. Risk factors of rejection in renal transplant recipients: A narrative review. J Clin Med. (2022) 11(5):1392. doi: 10.3390/jcm11051392 35268482 PMC8911293

[5] Konvalinka A Tinckam K. Utility of HLA antibody testing in kidney transplantation. J Am Soc Nephrol. (2015) 26:1489–502. doi: 10.1681/ASN.2014080837 PMC4483594 25804279

[6] Hemker HC Giesen P AlDieri R Regnault V de Smed E Wagenvoord R. The calibrated automated thrombogram (CAT): a universal routine test for hyper- and hypocoagulability. Pathophysiol Haemost Thromb. (2002) 32:249–53. doi: 10.1159/000073575 13679651

[7] Castoldi E Rosing J. Thrombin generation tests. Thromb Res. (2011) 127 Suppl 3:S21–5. doi: 10.1016/S0049-3848(11)70007-X 21262433

[8] Duarte RCF Ferreira CN Rios DRA Reis HJD Carvalho MDG. Thrombin generation assays for global evaluation of the hemostatic system: perspectives and limitations. Rev Bras Hematol Hemoter. (2017) 39:259–65. doi: 10.1016/j.bjhh.2017.03.009 PMC5568585 28830606

[9] Tripodi A. Thrombin generation assay and its application in the clinical laboratory. Clin Chem. (2016) 62:699–707. doi: 10.1373/clinchem.2015.248625 26955824

[10] Tripodi A. Usefulness of thrombin generation. Hamostaseologie. (2020) 40:509–14. doi: 10.1055/a-1200-0417 32731296

[11] Nemes B Barta A Ivadi G Karai B Szantho E Hevessy Z. T cell subset profile and appearance of donor-specific antibodies in primary and retransplanted kidney recipients. Transplant Proc. (2019) 51:1215–25. doi: 10.1016/j.transproceed.2019.04.002 31101201

[12] Lachmann N Todorova K Schulze H Schonemann C. Luminex((R)) and its applications for solid organ transplantation, hematopoietic stem cell transplantation, and transfusion. Transfus Med Hemother. (2013) 40:182–9. doi: 10.1159/000351459 PMC3725018 23922543

[13] Sablik KA Clahsen-van Groningen MC Looman CWN Damman J van Agteren M Betjes MGH. Treatment with intravenous immunoglobulins and methylprednisolone may significantly decrease loss of renal function in chronic-active antibody-mediated rejection. BMC Nephrol. (2019) 20:218. doi: 10.1186/s12882-019-1385-z 31200654 PMC6567552

[14] Roufosse C Simmonds N Clahsen-van Groningen M Haas M Henriksen KJ Horsfield C. A 2018 reference guide to the banff classification of renal allograft pathology. Transplantation. (2018) 102:1795–814. doi: 10.1097/TP.0000000000002366 PMC7597974 30028786

[15] Toth NK Szekely EG Czuriga-Kovacs KR Sarkady F Nagy O Lanczi LI. Elevated factor VIII and von willebrand factor levels predict unfavorable outcome in stroke patients treated with intravenous thrombolysis. Front Neurol. (2018) 8:721. doi: 10.3389/fneur.2017.00721 29410644 PMC5787073

[16] Loczi L Orban-Kalmandi R Arokszallasi T Fekete I Fekete K Heja M. Thrombin generation as a predictor of outcomes in patients with non-traumatic intracerebral hemorrhage. Front Neurol. (2022) 13:912664. doi: 10.3389/fneur.2022.912664 36061990 PMC9436391

[17] Orban-Kalmandi R Szegedi I Sarkady F Fekete I Fekete K Vasas N. A modified *in vitro* clot lysis assay predicts outcomes and safety in acute ischemic stroke patients undergoing intravenous thrombolysis. Sci Rep. (2021) 11:12713. doi: 10.1038/s41598-021-92041-1 34135389 PMC8208992

[18] Longstaff C subcommittee on f. Development of Shiny app tools to simplify and standardize the analysis of hemostasis assay data: communication from the SSC of the ISTH. J Thromb Haemost. (2017) 15:1044–6. doi: 10.1111/jth.13656 28304129

[19] Djamali A Kaufman DB Ellis TM Zhong W Matas A Samaniego M. Diagnosis and management of antibody-mediated rejection: current status and novel approaches. Am J Transplant. (2014) 14:255–71. doi: 10.1111/ajt.12589 PMC4285166 24401076

[20] Levitsky J Freifeld A Lyden E Stoner J Florescu D Langnas A. Evaluation of the coagulation and inflammatory responses in solid organ transplant recipients and donors. Clin Transplant. (2009) 23:943–50. doi: 10.1111/j.1399-0012.2009.01038.x 19624696

[21] Malyszko J Malyszko JS Hryszko T Mysliwiec M. Thrombin activatable fibrinolysis inhibitor in hypertensive kidney transplant recipients. Transplant Proc. (2006) 38:105–7. doi: 10.1016/j.transproceed.2005.11.072 16504676

[22] Mota AP Alpoim PN de Figueiredo RC Simoes e Silva AC Gomes KB Dusse LM. Hemostatic Parameters according to Renal Function and Time after Transplantation in Brazilian Renal Transplanted Patients. Dis Markers. (2015) 2015:472750. doi: 10.1155/2015/472750 26229221 PMC4502328

[23] Stallone G Pontrelli P Rascio F Castellano G Gesualdo L Grandaliano G. Coagulation and fibrinolysis in kidney graft rejection. Front Immunol. (2020) 11:1807. doi: 10.3389/fimmu.2020.01807 32983089 PMC7477357

[24] Pollinger HS Stegall MD Gloor JM Moore SB Degoey SR Ploeger NA. Kidney transplantation in patients with antibodies against donor HLA class II. Am J Transplant. (2007) 7:857–63. doi: 10.1111/j.1600-6143.2006.01699.x 17295642

[25] Freitas MC Rebellato LM Ozawa M Nguyen A Sasaki N Everly M. The role of immunoglobulin-G subclasses and C1q in *de novo* HLA-DQ donor-specific antibody kidney transplantation outcomes. Transplantation. (2013) 95:1113–9. doi: 10.1097/TP.0b013e3182888db6 23514959

[26] Van Loon E Lamarthee B Barba T Claes S Coemans M de Loor H. Circulating donor-specific anti-HLA antibodies associate with immune activation independent of kidney transplant histopathological findings. Front Immunol. (2022) 13:818569. doi: 10.3389/fimmu.2022.818569 35281018 PMC8904423

[27] Lynch RJ Platt JL. Accommodation in renal transplantation: unanswered questions. Curr Opin Organ Transplant. (2010) 15:481–5. doi: 10.1097/MOT.0b013e32833b9c25 PMC3085890 20613524

[28] Beland S Desy O Ung RV Vallin P Latulippe E Riopel J. Tacrolimus prevents von Willebrand factor secretion by allostimulated human glomerular endothelium. Am J Transplant. (2018) 18:2314–21. doi: 10.1111/ajt.14944 29790290

[29] Rios DR Mota AP Carvalho MG Fernandes AP Gomes KB Dusse LM. ADAMTS13 and von Willebrand factor assessment before and after kidney transplantation. Clin Chim Acta. (2011) 412:2353–4. doi: 10.1016/j.cca.2011.08.034 21910980

[30] Zietek Z. Endothelial markers: thrombomodulin and von willebrand factor and risk of kidney thrombosis after transplantation. Transplant Proc. (2021) 53:1562–9. doi: 10.1016/j.transproceed.2021.03.011 33892933

[31] Xiang Y Hwa J. Regulation of VWF expression, and secretion in health and disease. Curr Opin Hematol. (2016) 23:288–93. doi: 10.1097/MOH.0000000000000230 PMC5209751 26771163

[32] Lip GY Blann A. von Willebrand factor: a marker of endothelial dysfunction in vascular disorders? Cardiovasc Res. (1997) 34(2):255–65. doi: 10.1016/s0008-6363(97)00039-4 9205537

[33] Kanaji S Fahs SA Shi Q Haberichter SL Montgomery RR. Contribution of platelet vs. endothelial VWF to platelet adhesion and hemostasis. J Thromb Haemost. (2012) 10:1646–52. doi: 10.1111/j.1538-7836.2012.04797.x PMC3419786 22642380

[34] Manook M Kwun J Sacks S Dorling A Mamode N Knechtle S. Innate networking: Thrombotic microangiopathy, the activation of coagulation and complement in the sensitized kidney transplant recipient. Transplant Rev (Orlando). (2018) 32:119–26. doi: 10.1016/j.trre.2018.01.001 PMC6497150 29935708

[35] Al-Awadhi S Raynaud M Louis K Bouquegneau A Taupin JL Aubert O. Complement-activating donor-specific anti-HLA antibodies in solid organ transplantation: systematic review, meta-analysis, and critical appraisal. Front Immunol. (2023) 14:1265796. doi: 10.3389/fimmu.2023.1265796 37849755 PMC10577173

[36] Golshayan D Schwotzer N Fakhouri F Zuber J. Targeting the complement pathway in kidney transplantation. J Am Soc Nephrol. (2023) 34:1776–92. doi: 10.1681/ASN.0000000000000192 PMC10631604 37439664

[37] Heurich M McCluskey G. Complement and coagulation crosstalk - Factor H in the spotlight. Immunobiology. (2023) 228:152707. doi: 10.1016/j.imbio.2023.152707 37633063

[38] Gibson B Connelly C Moldakhmetova S Sheerin NS. Complement activation and kidney transplantation; a complex relationship. Immunobiology. (2023) 228:152396. doi: 10.1016/j.imbio.2023.152396 37276614

