# Supplementary material for: Increased thrombin generation in kidney transplant recipients with donor-specific antibodies directed against human leukocyte antigens
Source: Front Immunol. 2024 Oct 25;15:1407407. doi: 10.3389/fimmu.2024.1407407 (PMC11543428; doi:10.3389/fimmu.2024.1407407)
Supplement: Supplementary file 1 [file DataSheet1.pdf]

Supplementary Table 1. Correlations between thrombin generation parameters and renal graft function at baseline in patients without DSAs

				Proteinuria	
		**Serum creatinine (μmol/L)**	**Serum eGFR (mL/min/1.73 m^2)**	**Urine protein (mg/L)**	**Urine protein/creatinine (mg/mmol)**
Thrombin generation parameters	Lag time (min)	r=0.3848<br>95% CI: -0.1329 to 0.7375<br>p=0.1271	r= -0.2798<br>95% CI: -0.6788 to 0.2466<br>p=0.2740	r=0.1634<br>95% CI: -0.3579 to 0.6070<br>p=0.5273	r=0.0589<br>95% CI: -0.4465 to 0.5359<br>p=0.8218
	ETP (nM*min)	r=0.0282<br>95% CI: -0.4708 to 0.5135<br>p=0.9153	r=0.0159<br>95% CI: -0.4803 to 0.5045<br>p=0.9528	r=0.0611<br>95% CI: -0.4447 to 0.5374<br>p=0.8169	r= -0.2892<br>95% CI: -0.6842 to 0.2370<br>p=0.2594
	Peak thrombin (nM)	r= -0.0201<br>95% CI: -0.4960 to 0.4650<br>p=0.9389	r= -0.0348<br>95% CI: -0.5070 to 0.4534<br>p=0.8945	r=0.2739<br>95% CI: -0.2381 to 0.6668<br>p=0.2874	r=0.2089<br>95% CI: -0.3021 to 0.6266<br>p=0.4210
	Time to peak (min)	r=0.2300<br>95% CI: -0.2960 to 0.6490<br>p=0.3714	r= -0.1945<br>95% CI: -0.6269 to 0.3296<br>p=0.4510	r= -0.0114<br>95% CI: -0.5011 to 0.4837<br>p=0.9666	r=0.1340<br>95% CI: -0.3838 to 0.5877<br>p=0.6059

Spearman or Pearson correlation. CI, confidence interval; eGFR, estimated glomerular filtration rate; ETP, endogenous thrombin potential

Supplementary Table 2. Correlations between thrombin generation parameters and renal graft function at follow-up in patients without DSAs

				Proteinuria	
		**Serum creatinine (μmol/L)**	**Serum eGFR (mL/min/1.73 m^2)**	**Urine protein (mg/L)**	**Urine protein/creatinine (mg/mmol)**
Thrombin generation parameters	Lag time (min)	r=0.2919<br>95% CI: -0.2169 to 0.6760<br>p=0.2398	r= -0.2084<br>95% CI: -0.6246 to 0.3000<br>p=0.4066	r=0.0344<br>95% CI: -0.5182 to 0.5669<br>p=0.9065	r=0.3681<br>95% CI: -0.2186 to 0.7593<br>p=0.1939
	ETP (nM*min)	r= -0.1063<br>95% CI: -0.5461 to 0.3794<br>p=0.6746	r=0.0735<br>95% CI: -0.4073 to 0.5225<br>p=0.7717	r= -0.2075<br>95% CI: -0.6649 to 0.3630<br>p=0.4766	r= -0.2745<br>95% CI: -0.7027 to 0.2998<br>p=0.3423
	Peak thrombin (nM)	r= -0.0900<br>95% CI: -0.5345 to 0.3934<br>p=0.7223	r=0.0727<br>95% CI: -0.4080 to 0.5219<br>p=0.7743	r= -0.0887<br>95% CI: -0.5915 to 0.4637<br>p=0.7629	r= -0.1430<br>95% CI: -0.6261 to 0.4194<br>p=0.6258
	Time to peak (min)	r=0.1673<br>95% CI: -0.3383 to 0.5979<br>p=0.5071	r= -0.0884<br>95% CI: -0.5439 to 0.4073<br>p=0.7272	r= -0.1912<br>95% CI: -0.6652 to 0.3926<br>p=0.5072	r=0.2530<br>95% CI: -0.3362 to 0.6999<br>p=0.3787

Spearman or Pearson correlation. CI, confidence interval; eGFR, estimated glomerular filtration rate; ETP, endogenous thrombin potential

Supplementary Table 3. Correlations between thrombin generation parameters and renal graft function at baseline in patients with DSAs

				Proteinuria	
		**Serum creatinine (µmol/L)**	**Serum eGFR (mL/min/1.73 m^2)**	**Urine protein (mg/L)**	**Urine protein/creatinine (mg/mmol)**
Thrombin generation parameters	Lag time (min)	r=0.1732<br>95% CI: -0.2326 to 0.5277<br>p=0.3876	r= -0.1102<br>95% CI: -0.4797 to 0.2924<br>p=0.5842	r= -0.0501<br>95% CI: -0.5040 to 0.4254<br>p=0.8384	r=0.0969<br>95% CI: -0.3861 to 0.5383<br>p=0.6929
	ETP (nM*min)	r=0.3790<br>95% CI: -0.0130 to 0.6700<br>p=0.0512	r= -0.3420<br>95% CI: -0.6459 to 0.0555<br>p=0.0808	r=0.1943<br>95% CI: -0.2983 to 0.6052<br>p=0.4254	r= -0.0114<br>95% CI: -0.4745 to 0.4566<br>p=0.9630
	Peak thrombin (nM)	r=0.1005<br>95% CI: -0.3014 to 0.4721<br>p=0.6180	r= -0.0342<br>95% CI: -0.4187 to 0.3607<br>p=0.8655	r=0.0215<br>95% CI: -0.4486 to 0.4824<br>p=0.9301	r=0.0149<br>95% CI: -0.4539 to 0.4772<br>p=0.9517
	Time to peak (min)	r=0.2749<br>95% CI: -0.1291 to 0.6006<br>p=0.1653	r= -0.1993<br>95% CI: -0.5469 to 0.2068<br>p=0.3189	r= -0.0009<br>95% CI: -0.4649 to 0.4663<br>p=0.9971	r= -0.0314<br>95% CI: -0.4899 to 0.4406<br>p=0.8983

Spearman or Pearson correlation. CI, confidence interval; eGFR, estimated glomerular filtration rate; ETP, endogenous thrombin potential

Supplementary Table 4. Correlations between thrombin generation parameters and renal graft function at follow-up in patients with DSAs

				Proteinuria	
		**Serum creatinine (µmol/L)**	**Serum eGFR (mL/min/1.73 m^2)**	**Urine protein (mg/L)**	**Urine protein/creatinine (mg/mmol)**
Thrombin generation parameters	Lag time (min)	r=0.1525<br>95% CI: -0.2791 to 0.5328<br>p=0.4769	r=0.0600<br>95% CI: -0.3629 to 0.4625<br>p=0.7803	r= -0.0073<br>95% CI: -0.4486 to 0.4368<br>p=0.9749	r= -0.1913<br>95% CI: -0.5845 to 0.2747<br>p=0.4061
	ETP (nM*min)	r=0.5085<br>95% CI: 0.1419 to 0.7525<br>p=**0.0094**	r= -0.5073<br>95% CI: -0.7517 to -0.1403<br>p=**0.0096**	r=0.1699<br>95% CI: -0.2825 to 0.5605<br>p=0.4615	r=0.1732<br>95% CI: -0.2793 to 0.5628<br>p=0.4527
	Peak thrombin (nM)	r=0.4263<br>95% CI: 0.0374 to 0.7030<br>p=**0.0336**	r= -0.3696<br>95% CI: -0.6673 to 0.0299<br>p=0.0690	r=0.0874<br>95% CI: -0.3577 to 0.5003<br>p=0.7062	r=0.1356<br>95% CI: -0.3145 to 0.5359<br>p=0.5578
	Time to peak (min)	r=0.0613<br>95% CI: -0.3530 to 0.4555<br>p=0.7710	r=0.1157<br>95% CI: -0.3041 to 0.4978<br>p=0.5818	r=0.1438<br>95% CI: -0.3192 to 0.5514<br>p=0.5340	r= -0.1554<br>95% CI: -0.5596 to 0.3085<br>p=0.5011

Spearman or Pearson correlation. CI, confidence interval; eGFR, estimated glomerular filtration rate; ETP, endogenous thrombin potential

Supplementary Table 5. Histological characteristics of biopsies in DSA– and DSA+ patients

**Variable**	**DSA–**	**DSA+**
Number of individuals, *n*	2	13
Interstitial inflammation (*i*)		
0	2	13
1	0	0
2	0	0
3	0	0
Tubulitis (*t*)		
0	2	13
1	0	0
2	0	0
3	0	0
Intimal arteritis (*v*)		
0	2	13
1	0	0
2	0	0
3	0	0
Glomerulitis (*g*)		
0	2	4
1	0	3
2	0	1
3	0	5
Peritubular capillaritis (*ptc*)		
0	2	4
1	0	4
2	0	3
3	0	2
C4d		
0	2	8
1	0	3
2	0	2
3	0	0
Interstitial fibrosis (*ci*)		
0	0	1
1	2	8
2	0	4
3	0	0
Tubular atrophy (*ct*)		
0	0	1
1	2	8
2	0	4
3	0	0
Vascular fibrous intimal thickening (*cv*)		
0	0	2
1	2	8
2	0	3

3	0	0
Glomerular basement membrane double contours (*cg*)		
0	2	6
1	0	2
2	0	2
3	0	3
Mesangial matrix expansion (*mm*)		
0	1	5
1	0	7
2	1	0
3	0	1
Arteriolar hyalinosis (*ah*)		
0	0	2
1	1	4
2	1	5
3	0	2
Total inflammation (*ti*)		
0	2	12
1	0	1
2	0	0
3	0	0
Inflammation in the area of IFTA (*i-IFTA*)		
0	1	9
1	1	3
2	0	1
3	0	0
Primary rejection diagnosis		
No rejection	2	5
Active ABMR	0	1
Chronic active ABMR	0	7

Numbers indicate cases. ABMR, Antibody Mediated Rejection; DSA, donor specific antibodies

Supplementary Table 6. Correlations between thrombin generation parameters and various laboratory parameters at baseline in patients without DSAs

		Age (y)	hsCRP (mg/L)	Fibrinogen (g/L)	Platelet count (G/L)	VWF antigen (%)	FVIII activity (%)	sPsel (ng/mL)	sEsel (ng/mL)	C3 (g/L)	C4 (g/L)
Thrombin generation parameters	Lag time (min)	r=-0.1376<br>95% CI: -0.5901 to 0.3807<br>p=0.5961	r=0.2778<br>95% CI: -0.2487 to 0.6776<br>p=0.2782	r=0.2990<br>95% CI: -0.2674 to 0.7119<br>p=0.2768	r= -0.2170<br>95% CI: -0.6409 to 0.3085<br>p=0.3994	r=0.3268<br>95% CI: -0.1975 to 0.7057<br>p=0.1994	r=0.3641<br>95% CI: -0.1564 to 0.7263<br>p=0.1502	r=0.5350<br>95% CI: 0.0374 to 0.8200<br>p=**0.0347**	r=0.2948<br>95% CI: -0.2312 to 0.6875<br>p=0.2489	r=-0.0024<br>95% CI: -0.4943 to 0.4906<br>p=0.9943	r=-0.1873<br>95% CI: -0.6224 to 0.3362<br>p=0.4680
	ETP (nM*min)	r= -0.2059<br>95% CI: -0.6341 to 0.3189<br>p=0.4264	r=0.3350<br>95% CI: -0.1886 to 0.7103<br>p=0.1887	r=0.5143<br>95% CI: -0.0139 to 0.8181<br>p=0.0524	r= -0.1337<br>95% CI: -0.5875 to 0.3841<br>p=0.6068	r=0.2598<br>95% CI: -0.2668 to 0.6669<br>p=0.3127	r=0.1055<br>95% CI: -0.4082 to 0.5684<br>p=0.6852	r=0.3480<br>95% CI: -0.1743 to 0.7175<br>p=0.1712	r=0.0563<br>95% CI: -0.4485 to 0.5340<br>p=0.8315	r=0.4191<br>95% CI: -0.0924 to 0.7556<br>p=0.0954	r=0.2827<br>95% CI: -0.2437 to 0.6805<br>p=0.2695
	Peak thrombin (nM)	r=0.0359<br>95% CI: -0.4525 to 0.5078<br>p=0.8909	r=0.0414<br>95% CI: -0.4481 to 0.5119<br>p=0.8744	r=0.4838<br>95% CI: -0.0378 to 0.7982<br>p=0.0677	r=0.1421<br>95% CI: -0.3633 to 0.5829<br>p=0.5864	r=0.4422<br>95% CI: -0.0487 to 0.7611<br>p=0.0755	r=0.4875<br>95% CI: 0.0089 to 0.7844<br>p=**0.0471**	r=0.1143<br>95% CI: -0.3877 to 0.5639<br>p=0.6624	r=0.0804<br>95% CI: -0.4163 to 0.5402<br>p=0.7588	r=0.7781<br>95% CI: 0.4752 to 0.9161<br>p=**0.0002**	r=0.5836<br>95% CI: 0.1431 to 0.8311<br>p=**0.0139**
	Time to peak (min)	r= -0.3061<br>95% CI: -0.6939 to 0.2195<br>p=0.2307	r=0.0494<br>95% CI: -0.4541 to 0.5290<br>p=0.8509	r=0.4179<br>95% CI: -0.1365 to 0.7730<br>p=0.1218	r= -0.2571<br>95% CI: -0.6653 to 0.2695<br>p=0.3165	r=0.0909<br>95% CI: -0.4203 to 0.5584<br>p=0.7270	r= -0.2526<br>95% CI: -0.7310 to 0.3922<br>p=0.4253	r=0.2474<br>95% CI: -0.2977 to 0.6709<br>p=0.3529	r=0.1352<br>95% CI: -0.3827 to 0.5885<br>p=0.6024	r= -0.1868<br>95% CI: -0.6221 to 0.3366<br>p=0.4697	r= -0.2867<br>95% CI: -0.6828 to 0.2396<br>p=0.2619

Spearman or Pearson correlation. C3, complement component 3; C4, complement component 4; CI, confidence interval; ETP, endogenous thrombin potential; FVIII, factor VIII; hsCRP, high sensitivity C-reactive protein measurement; sPsel, soluble P selectin; sEsel, soluble E selectin; VWF, von Willebrand factor

Supplementary Table 7. Correlations between thrombin generation parameters and various laboratory parameters at follow-up in patients without DSAs

		Age (y)	hsCRP (mg/L)	Fibrinogen (g/L)	Platelet count (G/L)	VWF antigen (%)	FVIII activity (%)	sPsel (ng/mL)	sEsel (ng/mL)	C3 (g/L)	C4 (g/L)
Thrombin generation parameters	Lag time (min)	r= -0.1687<br>95% CI: -0.5989 to 0.3370<br>p=0.5033	r=0.1713<br>95% CI: -0.3346 to 0.6005<br>p=0.4968	r=0.2411<br>95% CI: -0.2852 to 0.6557<br>p=0.3485	r= -0.1025<br>95% CI: -0.5538 to 0.3954<br>p=0.6857	r= -0.0818<br>95% CI: -0.5392 to 0.4128<br>p=0.7469	r=0.0699<br>95% CI: -0.4227 to 0.5307<br>p=0.7826	r=0.2671<br>95% CI: -0.2424 to 0.6611<br>p=0.2840	r=0.0113<br>95% CI: -0.4697 to 0.4872<br>p=0.9642	r=0.0662<br>95% CI: -0.5167 to 0.6072<br>p=0.8289	r= -0.0483<br>95% CI: -0.5957 to 0.5297<br>p=0.8751
	ETP (nM*min)	r= -0.0550<br>95% CI: -0.5088 to 0.4227<br>p=0.8282	r=0.1870<br>95% CI: -0.3067 to 0.6014<br>p=0.4575	r=0.3015<br>95% CI: -0.2095 to 0.6832<br>p=0.2395	r=0.5121<br>95% CI: -0.0594 to 0.7901<br>p=**0.0298**	r=0.2397<br>95% CI: -0.2558 to 0.6355<br>p=0.3380	r=0.4526<br>95% CI: -0.0181 to 0.7591<br>p=0.0593	r=0.0812<br>95% CI: -0.4008 to 0.5281<br>p=0.7486	r=0.3130<br>95% CI: -0.1802 to 0.6805<br>p=0.2059	r=0.5935<br>95% CI: 0.0631 to 0.8624<br>p=**0.0325**	r=0.5949<br>95% CI: 0.0653 to 0.8630<br>p=**0.0320**
	Peak thrombin (nM)	r= -0.1040<br>95% CI: -0.5444 to 0.3814<br>p=0.6814	r=0.2851<br>95% CI: -0.2096 to 0.6637<br>p=0.2514	r=0.2481<br>95% CI: -0.2640 to 0.6511<br>p=0.3369	r=0.4067<br>95% CI: -0.0742 to 0.7342<br>p=0.0939	r=0.1997<br>95% CI: -0.2946 to 0.6097<br>p=0.4269	r=0.4679<br>95% CI: 0.0013 to 0.7672<br>p=0.0502	r= -0.0433<br>95% CI: -0.5001 to 0.4323<br>p=0.8646	r=0.2028<br>95% CI: -0.2917 to 0.6117<br>p=0.4197	r=0.5366<br>95% CI: -0.0204 to 0.8394<br>p=0.0587	r=0.3467<br>95% CI: -0.2526 to 0.7537<br>p=0.2459
	Time to peak (min)	r= -0.1435<br>95% CI: -0.5820 to 0.3597<br>p=0.5699	r=0.1931<br>95% CI: -0.3144 to 0.6148<br>p=0.4425	r=0.2538<br>95% CI: -0.2727 to 0.6634<br>p=0.3232	r= -0.0712<br>95% CI: -0.5316 to 0.4216<br>p=0.7788	r= -0.1798<br>95% CI: -0.6061 to 0.3268<br>p=0.4754	r= -0.1562<br>95% CI: -0.5905 to 0.3484<br>p=0.5361	r=0.2447<br>95% CI: -0.2648 to 0.6474<br>p=0.3277	r= -0.0454<br>95% CI: -0.5128 to 0.4427<br>p=0.8579	r= -0.1183<br>95% CI: -0.6393 to 0.4771<br>p=0.6994	r=0.0523<br>95% CI: -0.5268 to 0.5983<br>p=0.8664

Spearman or Pearson correlation. C3, complement component 3; C4, complement component 4; CI, confidence interval; ETP, endogenous thrombin potential; FVIII, factor VIII; hsCRP, high sensitivity C-reactive protein measurement; sPsel, soluble P selectin; sEsel, soluble E selectin; VWF, von Willebrand factor

Supplementary Table 8. Correlations between thrombin generation parameters and various laboratory parameters at baseline in patients with DSAs

Thrombin generation parameters		Age (y)	hsCRP (mg/L)	Fibrinogen (g/L)	Platelet count (G/L)	VWF antigen (%)	FVIII activity (%)	sPsel (ng/mL)	sEsel (ng/mL)	C3 (g/L)	C4 (g/L)
Lag time (min)		r=0.1074	r=0.1025	r= -0.0420	r= -0.2934	r= -0.0563	r=-0.0036	r=0.0227	r=0.1453	r=0.5038	r=0.4696
		95% CI: -0.2950 to 0.4775	95% CI: -0.2996 to 0.4737	95% CI: -0.4400 to 0.3698	95% CI: -0.6133 to 0.1092	95% CI: -0.4368 to 0.3413	95% CI: -0.3932 to 0.3869	95% CI: -0.3706 to 0.4092	95% CI: -0.2595 to 0.5067	95% CI: 0.1415 to 0.7471	95% CI: 0.0973 to 0.7266
		p=0.5938	p=0.6109	p=0.8419	p=0.1375	p=0.7802	p=0.9854	p=0.9102	p=0.4697	p=**0.0074**	p=**0.0135**
ETP (nM*min)		r= -0.2393	r=0.4344	r=0.4062	r=0.4415	r= -0.0726	r=0.1515	r=0.1801	r=0.2662	r=0.1927	r=0.1616
		95% CI: -0.5757 to 0.1663	95% CI: 0.0534 to 0.7050	95% CI: 0.0008 to 0.6969	95% CI: 0.0620 to 0.7094	95% CI: -0.4500 to 0.3267	95% CI: -0.2536 to 0.5114	95% CI: -0.2259 to 0.5328	95% CI: -0.1383 to 0.5945	95% CI: -0.2134 to 0.5420	95% CI: -0.2439 to 0.5190
		p=0.2293	p=**0.0236**	p=**0.0439**	p=**0.0212**	p=0.7187	p=0.4507	p=0.3687	p=0.1796	p=0.3356	p=0.4207
Peak thrombin (nM)		r= -0.2100	r=0.0732	r=0.0200	r=0.3667	r=0.2531	r=0.0992	r= -0.0940	r= -0.0024	r=0.3060	r=0.2447
		95% CI: -0.5547 to 0.1961	95% CI: -0.3261 to 0.4505	95% CI: -0.3887 to 0.4221	95% CI: -0.0273 to 0.6621	95% CI: -0.1520 to 0.5854	95% CI: -0.3025 to 0.4711	95% CI: -0.4670 to 0.3073	95% CI: -0.3922 to 0.3880	95% CI: -0.0954 to 0.6219	95% CI: -0.1607 to 0.5795
		p=0.2931	p=0.7165	p=0.9244	p=0.0599	p=0.2028	p=0.6223	p=0.6409	p=0.9904	p=0.1206	p=0.2187
Time to peak (min)		r=0.0191	r=0.1796	r=0.0426	r= -0.3031	r= -0.1884	r=0.0571	r=0.1146	r= -0.2171	r=0.2015	r=0.2406
		95% CI: -0.3738 to 0.4062	95% CI: -0.2264 to 0.5324	95% CI: -0.3692 to 0.4405	95% CI: -0.6199 to 0.0986	95% CI: -0.5389 to 0.2177	95% CI: -0.3405 to 0.4375	95% CI: -0.2884 to 0.4831	95% CI: -0.5598 to 0.1890	95% CI: -0.2047 to 0.5485	95% CI: -0.1650 to 0.5766
		p=0.9247	p=0.3701	p=0.8395	p=0.1243	p=0.3467	p=0.7770	p=0.5693	p=0.2766	p=0.3135	p=0.2268

Spearman or Pearson correlation. C3, complement component 3; C4, complement component 4; CI, confidence interval; ETP, endogenous thrombin potential; FVIII, factor VIII; hsCRP, high sensitivity C-reactive protein measurement; sPsel, soluble P selectin; sEsel, soluble E selectin; VWF, von Willebrand factor

Supplementary Table 9. Correlations between thrombin generation parameters and various laboratory parameters at follow-up in patients with DSAs

		Age (y)	hsCRP (mg/L)	Fibrinogen (g/L)	Platelet count (G/L)	VWF antigen (%)	FVIII activity (%)	sPsel (ng/mL)	sEsel (ng/mL)	C3 (g/L)	C4 (g/L)
Thrombin generation parameters	Lag time (min)	r=0.1196	r=0.4712	r=0.4968	r= -0.3527	r=0.0104	r=0.0579	r=0.3052	r= -0.0019	r=0.4392	r=0.5166
		95% CI: -0.3097 to 0.5084	95% CI: 0.0710 to 0.7406	95% CI: 0.0935 to 0.7600	95% CI: -0.6690 to 0.0717	95% CI: -0.4052 to 0.4226	95% CI: -0.3648 to 0.4608	95% CI: -0.1724 to 0.6666	95% CI: -0.4443 to 0.4412	95% CI: -0.0043 to 0.7384	95% CI: 0.0958 to 0.7808
		p=0.5778	p=**0.0201**	p=**0.0159**	p=0.0910	p=0.9612	p=0.7881	p=0.1907	p=0.9933	p=**0.0464**	p=**0.0165**
	ETP (nM*min)	r= -0.2962	r=0.2512	r=0.4970	r=0.3611	r=0.4388	r=0.3756	r=0.1573	r= -0.1406	r=0.2860	r=0.3802
		95% CI: -0.6249 to 0.1217	95% CI: -0.1598 to 0.5880	95% CI: 0.1171 to 0.7501	95% CI: -0.0396 to 0.6618	95% CI: 0.0528 to 0.7107	95% CI: -0.0229 to 0.6711	95% CI: -0.2944 to 0.5515	95% CI: -0.5307 to 0.2988	95% CI: -0.1542 to 0.6314	95% CI: -0.0493 to 0.6910
		p=0.1599	p=0.2258	p=**0.0135**	p=0.0761	p=**0.0282**	p=0.0643	p=0.4958	p=0.5327	p=0.1970	p=0.0809
	Peak thrombin (nM)	r= -0.1074	r=0.1384	r=0.4640	r=0.3898	r=0.4701	r=0.5894	r=0.2347	r= -0.0630	r=0.2183	r=0.2854
		95% CI: -0.4821 to 0.3004	95% CI: -0.2716 to 0.5058	95% CI: 0.0746 to 0.7307	95% CI: -0.0062 to 0.6802	95% CI: 0.0920 to 0.7297	95% CI: 0.2533 to 0.7986	95% CI: -0.2192 to 0.6051	95% CI: -0.4721 to 0.3683	95% CI: -0.2239 to 0.5860	95% CI: -0.1548 to 0.6311
		p=0.6092	p=0.5095	p=**0.0224**	p=0.0541	p=**0.0177**	p=**0.0019**	p=0.3058	p=0.7804	p=0.3291	p=0.1979
	Time to peak (min)	r= -0.0793	r=0.3085	r=0.1076	r= -0.2277	r= -0.3753	r= -0.3675	r=0.1080	r=0.1804	r=0.2499	r=0.3210
		95% CI: -0.4697 to 0.3370	95% CI: -0.1108 to 0.6346	95% CI: -0.3206 to 0.4993	95% CI: -0.5797 to 0.1959	95% CI: -0.6777 to 0.0356	95% CI: -0.6728 to 0.0446	95% CI: -0.3516 to 0.5256	95% CI: -0.2734 to 0.5685	95% CI: -0.2047 to 0.6158	95% CI: -0.1295 to 0.6616
		p=0.7061	p=0.1335	p=0.6168	p=0.2738	p=0.0645	p=0.0707	p=0.6413	p=0.4217	p=0.2621	p=0.1453

Spearman or Pearson correlation. C3, complement component 3; C4, complement component 4; CI, confidence interval; ETP, endogenous thrombin potential; FVIII, factor VIII; hsCRP, high sensitivity C-reactive protein measurement; sPsel, soluble P selectin; sEsel, soluble E selectin; VWF, von Willebrand factor
